# Quercetin Induces Apoptosis via Downregulation of Vascular Endothelial Growth Factor/Akt Signaling Pathway in Acute Myeloid Leukemia Cells

**DOI:** 10.3389/fphar.2020.534171

**Published:** 2020-12-10

**Authors:** Huan Shi, Xin-Yu Li, Yao Chen, Xing Zhang, Yong Wu, Zi-Xuan Wang, Pan-Hong Chen, Hui-Qi Dai, Ji Feng, Sayantan Chatterjee, Zhong-Jie Li, Xiao-Wei Huang, Hong-Qiao Wei, Jigang Wang, Guo-Dong Lu, Jing Zhou

**Affiliations:** ^1^ Department of Physiology, School of Preclinical Medicine, Guangxi Medical University, Nanning, China; ^2^ Department of Physiology, School of Medicine, Hunan University of Medicine, Huaihua, China; ^3^ Artemisinin Research Center and the Institute of Chinese Materia Medica, China Academy of Chinese Medical Sciences, Beijing, China; ^4^ Department of Toxicology, School of Public Health, Guangxi Medical University, Nanning, China; ^5^ Key Laboratory of High-incidence-Tumor Prevention and Treatment (Guangxi Medical University), Ministry of Education of China, Nanning, China; ^6^ Cancer Science Institute of Singapore, National University of Singapore, Singapore, Singapore

**Keywords:** quercetin, vascular endothelial growth factor/PI3K/Akt, mitochondria, apoptosis, acute myeloid leukemia

## Abstract

Acute myeloid leukemia (AML) is an aggressive haematological malignancy characterized by highly proliferative accumulation of immature and dysfunctional myeloid cells. Quercetin (Qu), one kind of flavonoid, exhibits anti-cancer property in multiple types of solid tumor, but its effect on acute myeloid leukemia is less studied, and the underlying mechanisms still largely unknown. This study aimed to explore the specific target and potential mechanism of quercetin-induced cell death in AML. First, we found that quercetin induces cell death in the form of apoptosis, which was caspase dependent. Second, we found that quercetin-induced apoptosis depends on the decrease of mitochondria membrane potential (MMP) and Bcl-2 proteins. With quantitative chemical proteomics, we observed the downregulation of VEGFR2 and PI3K/Akt signaling in quercetin-treated cells. Consistently, cell studies also identified that VEGFR2 and PI3K/Akt signaling pathways are involved in the action of quercetin on mitochondria and Bcl-2 proteins. The decrease of MMP and cell death could be rescued when PI3K/Akt signaling is activated, suggesting that VEGFR2 and PI3K/Akt exert as upstream regulators for quercetin effect on apoptosis induction in AML cells. In conclusion, our findings from this study provide convincing evidence that quercetin induces cell death via downregulation of VEGF/Akt signaling pathways and mitochondria-mediated apoptosis in AML cells.

## Introduction

Acute myeloid leukemia (AML) is the most common acute leukemia in adults. This disease is an aggressive hematological malignancy characterized by highly proliferative accumulation of immature and dysfunctional myeloid cells (Saultz and Garzon, 2016). Conventional clinical treatment of AML includes chemotherapy, radiotherapy and immunotherapy. Despite the therapeutic strategy has been significantly improved recently, acquisition of resistance in AML patients results in frequent recurrence and poor prognosis (Dohner et al., 2015; Dombret and Gardin, 2016). The unsatisfactory and limited efficacy of current therapy regimens suggests the need for alternative and target-specific drugs that may offer better clinical benefits.

Some growth factor pathways have been reported to show significant impacts on the development of AML, especially vascular endothelial growth factor (VEGF) could offer leukemia cells advantages on proliferation, survival, and chemotherapy resistance via both autocrine and paracrine manners (Dias et al., 2001; Papaemmanuil et al., 2016). VEGF is known as one of the families involved in the regulation of vascular angiogenesis (Lee et al., 2007). It has been reported that VEGF level is a prognostic factor of AML (Kampen et al., 2013; Papaemmanuil et al., 2016). More importantly, accumulating literatures have proven that aberrant regulation of VEGF signaling triggers the activation via PI3K/AKT pathway is related to the poor prognosis of AML (Zhang et al., 2015; Gong et al., 2019). Therefore, VEGF could be an important autocrine growth factor involved in AML progression and therapy resistance. Current studies demonstrated that exogenous VEGF can protect AML cells from chemotherapy-induced apoptosis (Jayson et al., 2016), and agents against VEGF/VEGFR pathway, including anti-VEGF antibody and small molecular tyrosine kinase inhibitors, have been introduced in leukemia therapy (Madlambayan et al., 2010).

Quercetin (Qu), one kind of flavonoid, is widely distributed in various vegetables and fruits, exhibiting a broad spectrum of biological effects, such as antioxidant, anti-inflammatory and anticancer (Murakami et al., 2008). In the last decade, the anticancer effects of quercetin alone or combination with other drugs have been proposed, and this pharmacological activity has been attributed to its effects on cell cycle arrest, apoptosis and angiogenesis via blocking VEGF/PI3K/Akt in prostate cancer cells (Sun et al., 2018) or ERK signaling pathways in colorectal cancer cells (Salama et al., 2019; Zhang et al., 2019). Nevertheless, the effect of quercetin in AML cells is less studied and the particular mechanisms have not been identified (Lee et al., 2015).

Due to the inhibitory effect of quercetin on VEGF/Akt signaling, and the potential of VEGF/VEGFR as therapeutic target in AML, we hypothesized that quercetin may exert anti-cancer effect via VEGF/Akt signaling in AML. In this study, we have certified that quercetin induces mitochondria-dependent apoptosis in AML cells, which is associated with the involvement of Bcl-2 proteins and the inhibition on VEGFR2 and PI3K/Akt signaling pathways.

## Materials and Methods

### Cell Lines and Cell Culture

Human myeloid leukemia HL-60 cells, MV4-11 cells purchased from National Infrastructure of Cell Line Resource were maintained in Roswell Park Memorial Institute (RPMI) 1640 Medium (Gibco) and Iscove's Modified Dulbecco's Medium (IMDM, Gibco). Both culture media were supplemented with 10% Fetal bovine serum (FBS), 1% streptomycin/penicillin (Beyotime). Cell lines were maintained at 37°C in a humidified atmosphere containing 5% CO2.

### Antibodies and Reagents

Quercetin (Qu, Q4951), Propidium iodide (PI, P4170), Bafilomycin-A1 (Baf-A1, 19-148), Chloroquine (CQ, C6628) were purchased from Sigma-Aldrich (St. Louis, MO). Lyso-Tracker Red DND-99 (L7528) was obtained from Invitrogen (St. California), SAR405 (S7682) was ordered from Selleck (St. Houston, TEX), dimethyl sulfoxide (DMSO) was purchased from MP Biomedicals. Z-IETD-FMK (HY-101297) and Z-VAD-FMK (HY-16658B) were purchased from MCE, Z-LEHD-FMK (B3233) was provided by APEXBIO. Human IGF-1 (100-11-100), Human VEGF (100-20-10) were purchased from PeproTech, insulin (Human Recombinant) was obtained from YEASEN. The primary antibodies against poly (ADP-ribose) polymerase (PARP, #9532), Caspase 8 (#9746), Caspase 9 (#9502), Caspase 3 (#9668), Bid (#2002), Bax (#5023), pS6K (T389, #9234), pS6 (S235/236, #2211), p4EBP1 (T37/46, #2855), VEGFR2 (#9698), pVEGFR2 (Tyr1175, #3770), AKT (#4691), pAKT (S473, #4060), pS6K (T389, #9234), p4EBP1 (#2855), COX IV (#4850), Cytochrome C (#11940), Anti-Rabbit IgG (H + L), F (ab’)2 Fragment (Alexa Fluor^®^ 488 Conjugate) (#4412), HRP labeled secondary antibodies to rabbit (#7074) and mouse (#7076) were purchased from Cell Signaling Technology (Danvers, MA). α-tubulin (T5168) and microtubule-associated protein 1 light chain 3 (LC3, L7543) from Sigma-Aldrich (St. Louis, MO), p62 (PM045) from MBL (Nagoya, Japan), PI3K (ab191606), pPI3K (ab182651) were obtained from Abcam (Cambridge, United Kingdom). Bcl-2 (492), Mcl-1 (819) were obtained from Santa Cruz (St. California).

### Cell Viability Measurement

The CCK-8 assay was used to measure cell viability. According to the manufacturer’s protocol (Dojindo), cells were cultured in a 96-well plate (2 × 10^5^ cells/mL) and subjected to various concentrations of quercetin as indicated. Subsequently, 10 μl of CCK-8 was added to each well, and the plates were incubated at 37°C for 3 h. The absorbance at 450 nm was measured by using a microplate reader MuLTISKAN GO (Thermo Fisher).

### Propidium Iodide Staining

Cells were harvested and washed twice with ice-cold PBS, then the cell suspension was incubated in PI (5 μg/ml), and the fluorescence intensities of 10, 000 cells/sample were measured by flow cytometer (BECKMAN COULTER, South Carolina) at an excitation wavelength of 488 nm. Fluorescence intensity of PI were recorded via FL-2 channel.

### Hoechst Staining

At the end of indicated treatments, cells were fixed with methanol and stained with Hoechst solution (1 μg/ml) for 30 min at room temperature, then cells were washed twice with PBS. Nuclear staining was captured with a fluorescence microscope.

### Lactate Dehydrogenase Release Assay

The extent of cell death was detected using the cytotoxicity LDH assay kit (Dojindo). According to the manufacturer’s protocol, cells were seeded in 24-well plates (5 × 10^5^ cells/ml) and cultured in medium, after 24 h, the culture medium from each condition was transferred 100 μl to a 96-well plates for the quantification of LDH. The absorbance of each sample was read with a microplate reader at 490 nm.

### Measurement of Mitochondrial Membrane Potential

According to the manufacturer’s protocol of JC-1 or TMRM assay kit (Beyotime). MV4-11 or HL-60 cells (10^6^/ml) were resuspended in IMDM supplemented with 1% FBS and stained with 2.5 μg/ml JC-1 or 0.5 ml TMRM. After incubation at room temperature for 20 min, cells were washed twice with PBS and resuspended in 400 μl PBS for flow cytometry.

### Isolation of Mitochondrial and Cytosolic Proteins

The method was used as previously reported (Dimauro et al., 2012). Cells were collected by centrifugation at 200 *g* for 7 min, then resuspended in 200 μl of STM buffer (250 mM sucrose, comprising 250 mM sucrose, 50 mM Tris-HCl pH 7.4, 5 mM MgCl_2_, protease and phosphatase inhibitor cocktails), After passing through gauge #27 needle for 20 times to break the cell membrane, the cell homogenates were applied to a series of centrifugation at 50 *g* for 10 min, 500 g for 20 min and 15,000 g for 20 min to fractionate unbroken cells, heavy nuclear fraction and mitochondria fraction, respectively. Mitochondria fraction were resuspended in 20 μl lysis buffer (50 mM Tris HCl pH 6.8, 1 mM EDTA, 0.5% Triton-X-100, protease and phosphatase inhibitors). After the final centrifugation at 100, 000 g for 30 min, the supernatant was collected as the cytosol fraction. Equal amounts of mitochondrial and cytosolic protein were subjected to Western blotting.

### Enzyme-Linked Immunosorbent Assay Assay

Conditioned medium was prepared as mentioned above and VEGF level in the medium was determined using a commercial Human VEGF Quantikine ELISA kit (R&D Systems) according to the manufacturer’s instructions. The absorbance at 450 nm was measured on a microplate reader.

### Lysotracker Red Staining

Cells were incubated with 50 nM LysoTracker Red DND-99 (Invitrogen) for 30 min at 37°C after designed treatment. Stained cells were washed and resuspended with PBS. Fluorescence intensities of 10, 000 cells per sample were measured by flow cytometry at an excitation wavelength of 577 nm. We recorded the fluorescence of lysotracker Red using the FL-2 channel.

### Measurement of Cell Surface Expression Levels of VEGFR2

After designated treatments, cells in 6-well plates were collected and washed with PBS, then incubated with 100 μl staining buffer containing saturating amounts of anti-VEGFR2 antibody at room temperature for 1 h. After incubation, cells were washed twice with staining buffer and incubated with Alexa Fluor® 488 Conjugate secondary antibody for another 30 min. The VEGFR2 expression was analyzed with flow cytometer (BECKMAN COULTER).

### Western Blotting Analysis

At the end of designated treatments, cells were lysed in whole cell lysis buffer. After determination of protein concentration using BCA protein assay (Beyotime), equal amounts of protein were subjected to SDS-PAGE gels and transferred to PVDF membranes (Bio-Rad).The membrane was blocked with 5% nonfat milk in Tris-buffered saline with Tween 20 (TBST) for 1 h, then incubated with various primary antibodies and Secondary antibodies. The membrane was developed with the enhanced chemiluminescence method and detected using EVOS™ FL Auto Imaging System (Thermo Fisher).

### Isobaric Tag for Relative and Absolute Quantification Labeling and LCMS/MS Analyses

The iTRAQ labeling method was applied to investigate the proteome changes after quercetin treatment for 12 h according to manufacturer’s instructions and as described previously (Wang et al., 2016). Briefly, after protein digestion, the peptides were labeled with four respective isobaric tags for 2 h and then pooled together. The contaminants were removed by an iTRAQ Method Development Kit (SCIEX, 4352160) using the strong cation exchange chromatography technique. Dried samples were reconstituted with diluent of 2% acetonitrile and 0.05% formic acid. After using an Eksigent NanoLCUltra system coupled to the cHiPLCNanoflex system (Eksigent, United States), the iTRAQ labeled peptides were detected by MS/MS with a TripleTOF 5600 system (SCIEX) set and identified by the Paragon algorithm with Protein Pilot TM Software 4.5 (SCIEX).

### Statistical Analysis

Data were presented as mean ± S.D from three independent experiments. Kruskal-Wallis test, one-way analysis of variance (ANOVA) or Mann-Whitney test were applied for statistical analysis with GraphPad Prism 8 Software. A *p*-value < 0.05 was considered as statistically significant.

## Results

### Quercetin Promotes Cell Death in Both Acute Myeloid Leukemia Cells

**FIGURE 1:**
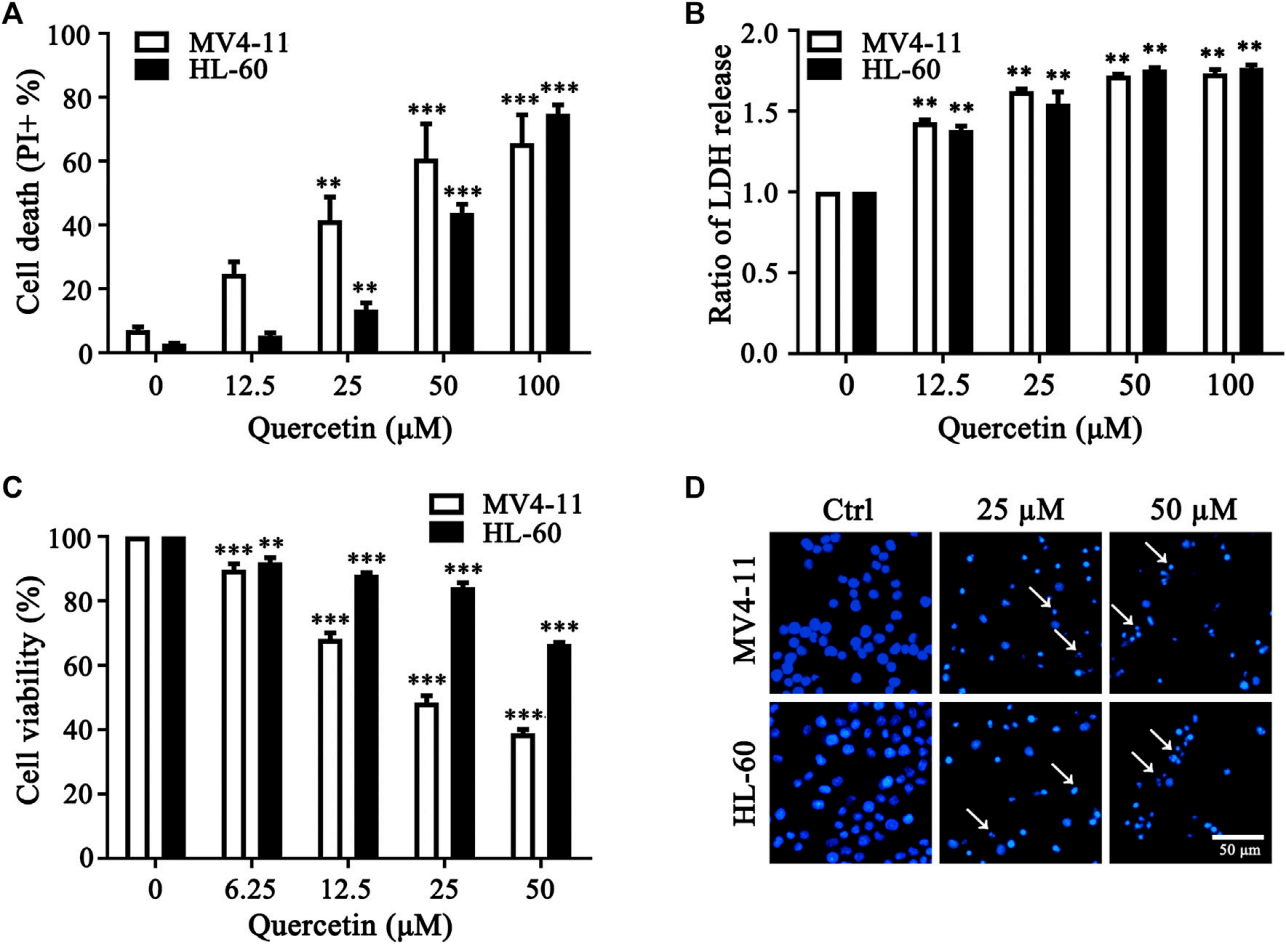
Quercetin promotes cell death in AML cells. **(A)** MV4-11, HL-60 cells were treated with quercetin for 24 h at various concentrations. Propidium Iodide (PI) positive cells were quantified by flow cytometer. The data are presented as the mean ± SD; *n* = 3; **p < 0.01, ***p < 0.001 versus the ctrl group. **(B)** Quercetin induced LDH release. Cells were treated as indicated in panel **(A)**, then cell death was quantitatively assessed by LDH release assay, ratios of LDH activity in individual group were normalized by control group are shown. The data are presented as the mean ± SD, *n* = 3, **p < 0.01, ***p < 0.001 versus the control group. **(C)** MV4-11 and HL-60 Cells were seeded in 96 well plates, and effects of various doses of quercetin on cell viability of both AML cell lines were evaluated by Cell Counting Kit-8 (CCK8). CCK8 was added 3 h before termination of the experiments and the absorbance was measured at 450 nm. The data are presented as the mean ± SD; *n* = 3; ***p < 0.001 versus the 0 μM group. **(D)** Effect of quercetin on nuclear morphological change of MV4-11 and HL-60 cells. Typical morphological changes were observed through fluorescence microscopy (×50) after Hoechst staining, and typical nuclear condensations are indicated by arrows.

To determine the anticancer effect of quercetin in acute myeloid leukemia (AML) cells, we utilized MV4-11 and HL-60, two myeloid leukemia cell lines to expose with different concentrations of quercetin. Notably, quercetin induced AML cell death in a dose-dependent manner (Figure 1A). As reported by literature, dead cells release the cytosolic lactate dehydrogenase (LDH) into culture medium due to plasma membrane permeabilization as a cell death marker (Kabakov and Gabai, 2018). Therefore, we also detected the release of LDH, and our result indicated that quercetin treatment induced LDH release in a dose-dependent manner in both AML cell lines (Figure 1B). To further determine if quercetin could inhibit the proliferation of AML cells, MV4-11 and HL-60 cells were cultured and treated with different doses of quercetin for 24 h by Cell Counting Kit-8 (CCK8), respectively. Consistently, quercetin could inhibit the proliferation of both MV4-11 and HL-60 cells (Figure 1C). As shown in Figure 1D, Hoechst staining results also suggested that quercetin is able to induce apoptotic cell death in AML cells. These results collectively indicated that quercetin promotes apoptotic cell death in AML cells.

### Quercetin Induces Caspase-Dependent Apoptosis in Acute Myeloid Leukemia Cells

**FIGURE 2:**
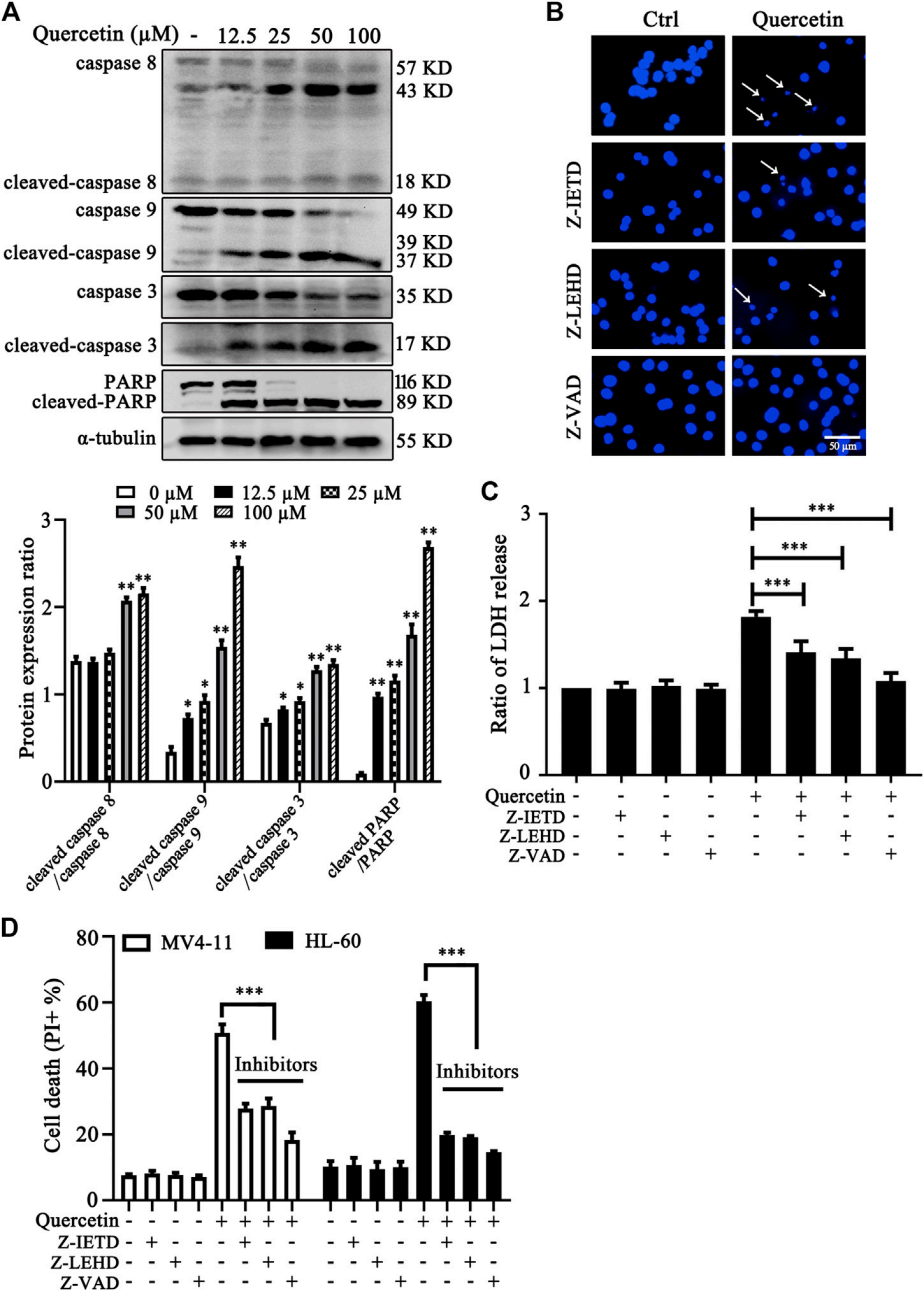
Quercetin induces caspase-dependent apoptosis in AML cells. **(A)** Quercetin activated caspase-cascade. MV4-11 cells were treated with quercetin for 24 h. Expression levels of apoptotic proteins were analyzed by western blotting. The relative densities of respective proteins were analyzed with Image J and presented with bar chart in lower panel. **(B-D)** Cell death can be blocked by specific caspase inhibitors. Cells were pretreated with Z-IETD-FMK (Z-IETD, 25 µM), Z-LEHD-FMK (Z-LEHD, 25 µM) or Z-VAD-FMK (Z-VAD, 40 µM) for 1 h, followed by the treatment of quercetin (25 µM) for 24 h, typical morphological changes were observed through fluorescence microscopy (×50) after Hoechst staining **(B)**; ratios of LDH activity in individual group were normalized by control group **(C)**; and cell death was quantified by flow cytometry after combined treatment on both MV4-11and HL-60 cells **(D)**. The data are presented as the mean ± SD; *n* = 3; ***p < 0.001 versus the quercetin group.

To investigate the involvement of caspase activation in quercetin-induced apoptosis, we first examined caspases activation and cleavage of poly ADP-ribose polymerase (PARP) after quercetin treatment. The results clearly showed that quercetin cleaves caspase 8, caspase 9, caspase 3 and PARP in dose-dependent manner on MV4-11 cells (Figure 2A). Next, to determine the contribution of these caspases to the quercetin-induced apoptosis in MV4-11 cells, Z-IETD-FMK (a caspase-8 inhibitor), Z-LEHD-FMK (a caspase-9 inhibitor) and Z-VAD-FMK (a general caspase inhibitor) were utilized to evaluate their protective effect on quercetin-induced apoptosis. The pretreatment with Z-IETD-FMK, Z-LEHD-FMK or Z-VAD-FMK could effectively block the apoptotic cell death induced by quercetin (Figure 2B), as well as the release of LDH (Figure 2C). Consistent with this observation, the PI exclusion assay also suggested that various caspase inhibitors show protective effect on both MV4-11 and HL-60 cells from quercetin-induced cell death (Figure 2D). Data from this set of experiments demonstrated that quercetin-induced apoptosis involves caspases activation and the mitochondria-dependent pathway.

### Quercetin Induces Apoptosis via Mitochondrial Pathway

**FIGURE 3:**
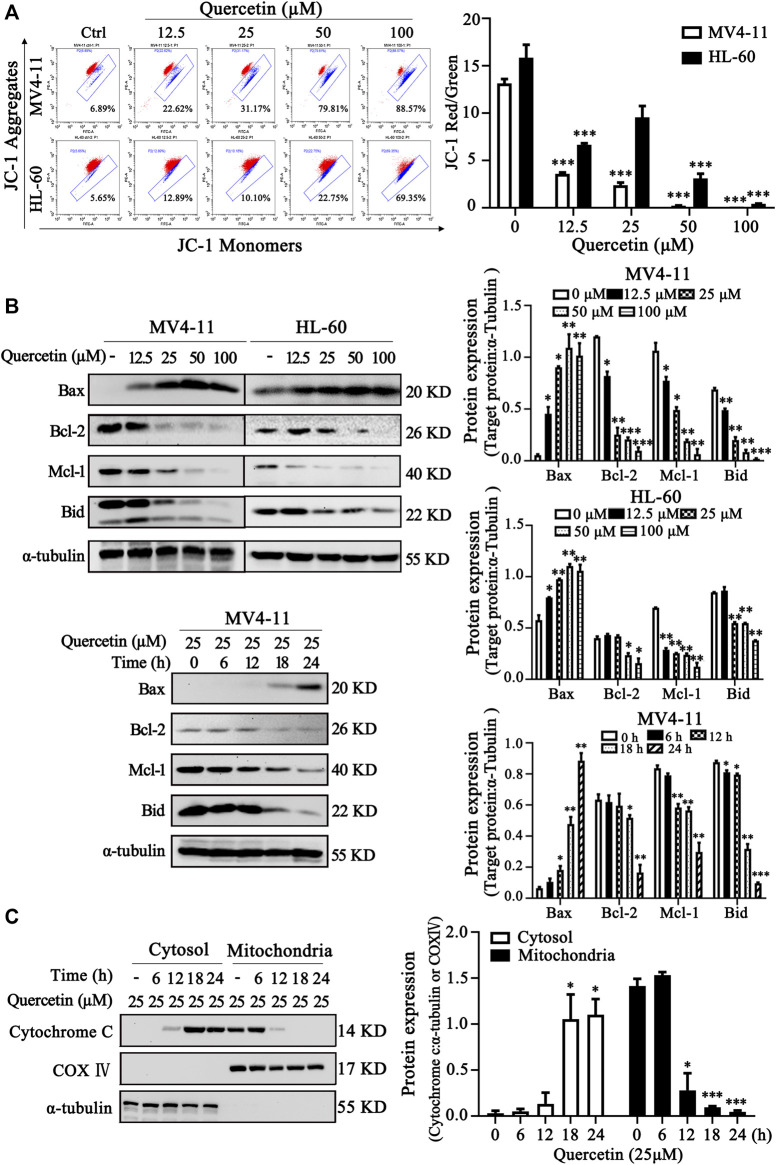
Quercetin induces apoptosis via mitochondrial pathway. **(A)** Quercetin caused Mitochondrial membrane potential (MMP) disruption. After indicated treatments, MV4-11 and HL-60 cells were stained with JC-1 dye. The ratio of red/green fluorescence is used to indicate MMP levels. The distribution rates in two AML cell lines are showed and cellular MMP levels were determined by flow cytometer. The results are presented as the mean ± SD; *n* = 3; ***p < 0.001 versus control group. **(B)** MV4-11 and HL-60 cells were treated with quercetin for indicated time courses or various concentrations, then cell lysates were collected and subjected to western blotting with various antibodies. The relative densities of respective proteins were analyzed with Image J and presented with bar chart in right panel. **(C)** MV4-11 cells were treated with 25 µM quercetin for indicated periods, while mitochondrial and cytoplasmic fractions were prepared as described in “*Materials and Methods*” section. The mitochondrial and cytoplasmic levels of cytochrome C were presented by Western blotting. The relative densities of respective proteins were analyzed with Image J and presented with bar chart in right panel. α-tubulin and COX-IV antibodies were used as cytoplasmic and mitochondrial markers, respectively.

It has been well established that the changes of mitochondrial membrane potential (MMP) and Bcl-2 family proteins play critical role in regulation of mitochondrial apoptotic pathway (Prenek et al., 2017). To further confirm the crucial role of mitochondrial pathway in quercetin-induced apoptosis, we examined MMP and Bcl-2 family protein levels in MV4-11 and HL-60 cells after quercetin treatment. After treatment with different dose quercetin for 10 h, we first observed the dose-dependent decreased of MMP in both AML cell lines (Figure 3A). More importantly, as shown in Figure 3B, quercetin promoted dose- and time-dependent decreases of full-length Bid and anti-apoptotic Bcl-2 family proteins (Mcl-1 and Bcl-2), together with the increase of pro-apoptotic Bcl-2 protein (Bax). It has been well studied that the consequence of MMP reduction and the involvement of Bcl-2 proteins is the release of cytochrome C from mitochondria to the cytoplasm (Cory and Adams, 2002). As expected, the time-dependent release of cytochrome C from mitochondria to cytosol was observed in quercetin-treated cells (Figure 3C). Therefore, these results suggest the involvement of Bcl-2 family proteins and mitochondrial pathway in quercetin-induced cell death.

### Quercetin Suppresses VEGFR2 and PI3K/Akt Signaling Pathway

**FIGURE 4:**
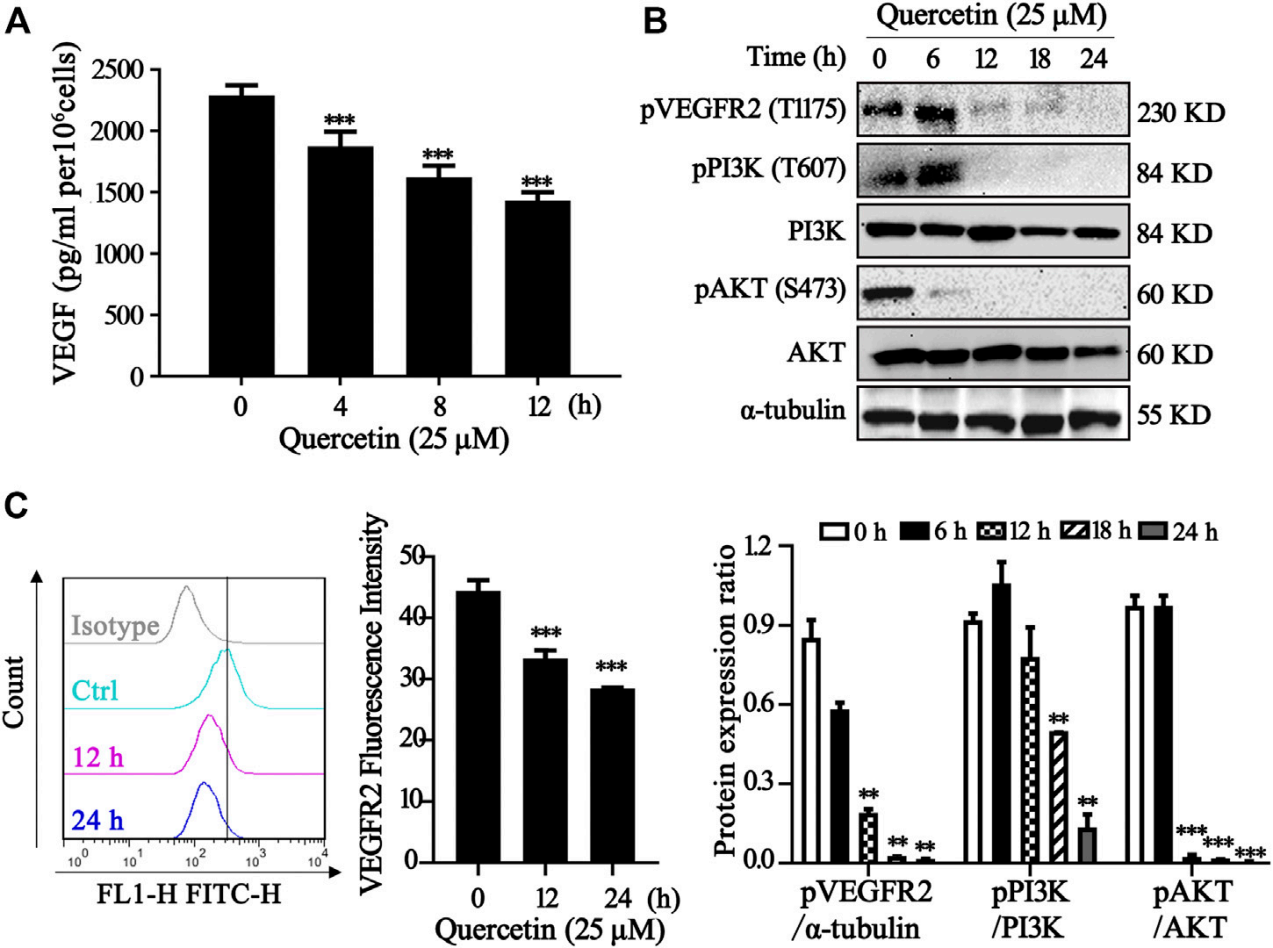
Quercetin suppresses VEGFR2 and PI3K/Akt signaling pathway. **(A)** Quercetin inhibited VEGF secretion in MV4-11 cells. Cells were treated with quercetin at 25 µM for 0 h, 4 h, 8 h, 12 h, then VEGF level was evaluated by ELISA. The results are presented as the mean ± SD; *n* = 3; ***p < 0.001 versus the control (0 µM) group. **(B)** Quercetin inhibits the activation of VEGFR2 and PI3K/Akt signaling pathway. MV4-11 cells were treated with quercetin at 25 µM for 0 h, 6 h, 12 h, 18 h, or 24 h, and cell lysates were collected followed by western blotting to detect the expression of proteins as indicated. α-tubulin served as loading control. The relative densities of respective proteins were analyzed with Image J and presented with bar chart in lower panel. **(C)** Cell surface expression levels of VEGFR2 were analyzed by flow cytometry after staining with anti-VEGFR2 antibody. The results are presented as the mean ± SD; *n* = 3; ***p < 0.001 versus ctrl group.

One important underlying mechanism of AML is the increased microvessel density and elevated VEGF and KDR expression levels (Rashika et al., 2016; Lin et al., 2019) Since VEGF exerts its effects mainly through two high-affinity tyrosine kinase receptors, VEGF receptor 1 (VEGFR1) and VEGFR2, the expression level of VEGF receptor in acute leukemia is a prognostic factor that correlates with disease progression and patient overall survival (Torres Sánchez et al., 2009; Wang et al., 2015). Inhibition of VEGF-VEGFR2 pathway could be a feasible therapeutic approach via repression on metastasis, proliferation and induction of apoptosis in AML cells (Verheul and Pinedo, 2000). In our study, we measured the VEGF level in MV4-11 cells after quercetin treatment at various time points. As shown in Figure 4A, treatment with quercetin inhibited VEGF secretion in a time-dependent manner in MV4-11 cells. To further validate the identified proteins as the direct binding targets of quercetin, iTRAQ-based quantitative chemical proteomics was performed to identify the targets of quercetin by using the quercetin probe. In our study, proteomic data showed that the expressions of Akt, MAPK and their downstream proteins were downregulated after quercetin treatment (Table 1).

**TABLE 1:** List of differentially expressed proteins in quercetin-treated MV4-11 cells.

Name	% Cov	Peptides	Qu1 VS ctrl1	Qu2 VS ctrl1	Qu1 VS ctrl2	Qu2 VS ctrl2	Ave ratio	*p*-value
MAPKAPK3	29.6	3	0.68	0.87	0.82	0.74	0.77	0.002
AKT1	51.9	3	0.69	0.88	0.97	0.77	0.82	0.031
RAC1	55.7	11	0.7	0.92	0.8	0.77	0.79	0.005
MAPK3	38.3	5	0.7	0.61	0.74	0.77	0.7	0.000437
MAP2K1	51.7	8	0.73	0.82	0.8	0.82	0.79	0.0002
PLA2G4A	25.1	5	0.74	0.95	0.9	0.82	0.85	0.019
MAPKAPK2	34	3	0.78	0.93	0.87	0.88	0.86	0.005
HSPB1	68.3	16	0.78	0.90	0.82	0.88	0.84	0.001

MAPKAPK3, MAP kinase-activated protein kinase 3; AKT1, RAC-alpha serine/threonine-protein kinase; RAC1, Ras-related C3 botulinum toxin substrate 1; MAPK3, Mitogen-activated protein kinase 3; MAP2K1, MAP kinase -activated protein kinase 2; PLA2G4A, Cytosolic phospholipase A2; MAPKAPK2, MAP kinase activated protein kinase 2; HSPB1, Heat shock protein beta-1. The representative proteins identified to be downregulated by quercetin in MV4-11 cells, and ratios indicated the protein changes after quercetin treatment. Two quercetin-probes (Qu1 and Qu2) and two DMSO-treated samples (ctrl1 and ctrl2) were analyzed as biological replicates. MV4-11 cells were incubated with 25 µM quercetin-probes or DMSO (negative control) for 4 h. The labeled samples were then pooled together and analyzed by LC-MS/MS to identify and quantify the target proteins, p-values less than 0.05 were considered statistically reliable hits.

The various downstream targets of VEGFR2, PI3K/Akt signaling pathway are important carcinogenic pathway that involved in the tumorigenesis of various cancers (Sun et al., 2018). In our study, Western blotting showed that the phosphorylation levels of Akt, PI3K, VEGFR2 were markedly impaired in the quercetin-treated cells compared with control group, while the total Akt, PI3K remained the same (Figure 4B). As shown in the Figure 4C, treatment with quercetin reduced VEGFR2 in a time-dependent manner. Our results are consistent with previous findings (Pratheeshkumar et al., 2012). Therefore, quercetin could suppress VEGFR2 and PI3K/Akt signaling pathway in MV4-11 cells.

### Involvement of VEGFR2 and PI3K/Akt Signaling Pathways in Quercetin-Induced Apoptosis

**FIGURE 5:**
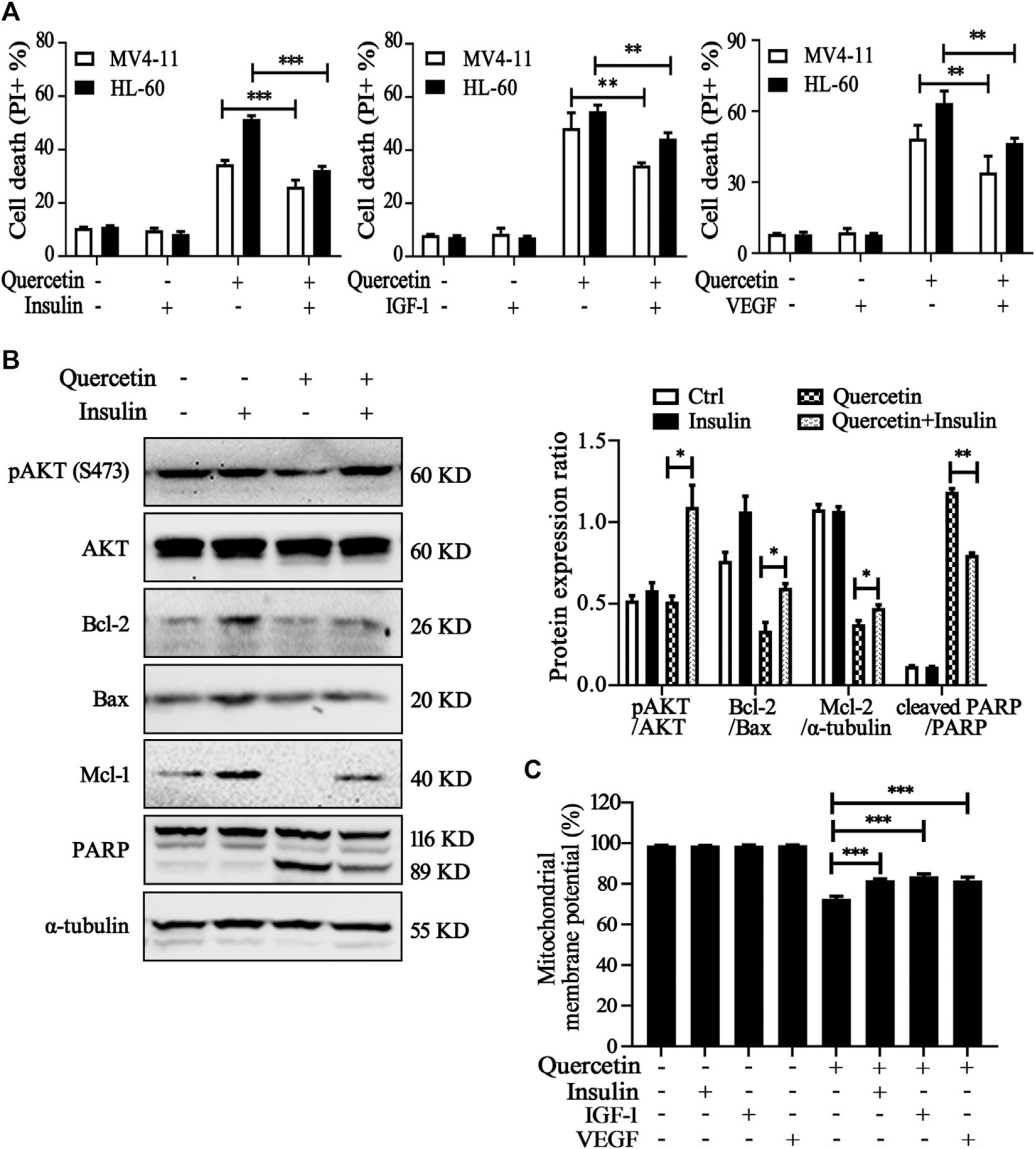
Involvement of VEGFR2 and PI3K/Akt signaling pathways in quercetin-induced apoptosis. **(A)** MV4-11 and HL-60 cells were pretreated with insulin (100 nM), IGF-1 (100 ng/ml) or VEGF (20 ng/ml) for 1 h, followed by quercetin treatment (25 μM for MV4-11, 50 μM for HL-60) for 16 h, cell death was determined by PI staining and flow cytometry. The data are presented as the mean ± SD; *n* = 3; **p < 0.01, ***p < 0.001 versus the quercetin group. **(B)** MV4-11 cells were pretreated with insulin (100 nM) for 1 h, then treated with quercetin (25 µM) for 12 h, at the end of treatment, cell lysates were collected and subjected to western blotting with the indicated antibodies. The relative densities of respective proteins were analyzed with Image J and presented with bar chart in right panel. **(C)** MV4-11 cells were pretreated with insulin (100 nM), IGF-1 (100 ng/ml) or VEGF (20 ng/ml) for 1 h, followed by quercetin (25 μM) for 10 h, Mitochondrial membrane potential (MMP) levels were monitored by TMRM through using flow cytometer.

Further experiments were conducted to elucidate the involvement of VEGFR2 and PI3K/AKT signaling pathways on quercetin-induced apoptosis. We treated MV4-11 and HL-60 cells with quercetin in the presence of insulin/IGF-1 (acting as AKT activator) or VEGF.Compared with quercetin single administration, combined treatment with insulin/IGF-1 or VEGF significantly reduced the population of apoptotic cell in both AML cell lines (Figure 5A). We further confirmed the protective effects of insulin on quercetin-treated MV4-11 cells, by the measurement on protein expression levels of pAkt, PARP, and Bcl-2 family members (Bcl-2, Mcl-1, and Bax). First of all, insulin treatment effectively prevented the suppression on Akt phosphorylation by quercetin; moreover, treatment with insulin prevented the decrease of anti-apoptotic proteins (Bcl-2 and Mcl-1), as well as blocked the cleavage of PARP induced by quercetin in MV4-11 cells (Figure 5B). As shown in Figure 5C, the reduction of MMP by quercetin was also blocked by insulin/IGF-1 and VEGF treatment, indicating VEGFR-Akt signaling acts at the upstream of mitochondria-dependent apoptosis which is triggered by quercetin.

### Quercetin-Mediated Autophagy Induction Confers Protection from Quercetin-Induced Apoptosis

**FIGURE 6:**
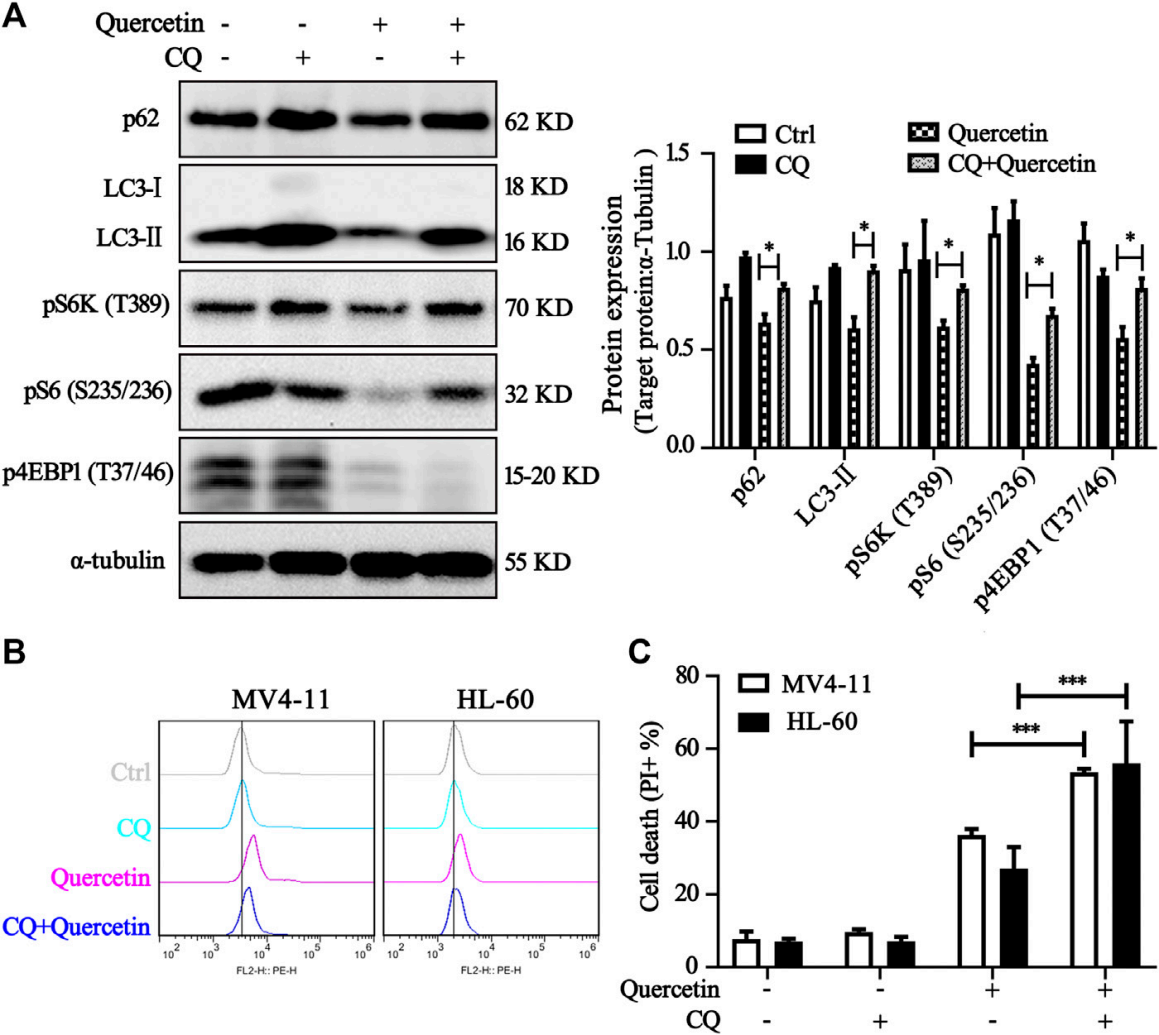
Quercetin-mediated autophagy induction confers protection from quercetin-induced apoptosis. **(A)** Quercetin induced autophagy in AML cells. MV4-11 cells were pretreated with chloroquine (CQ 20 μM) for 1 h, followed by quercetin (25 μM) for 4 h, cell lysates were collected and subjected to western blotting with the indicated antibodies. The relative densities of respective proteins were analyzed with Image J and presented with bar chart in right panel. **(B)** Activation of lysosomal function in cells treated with quercetin. MV4-11 and HL-60 cells were pretreated with or without CQ (20 μM) for 1 h, treated with quercetin (25 µM) for another 4 h. Cells were stained with LysoTracker Red DND-99 (50 nM) for 15 min, and fluorescence intensity of 10000 cells per sample was measured by flow cytometer. **(C)** Inhibition of autophagy enhanced quercetin-induced apoptosis. MV4-11 and HL-60 cells were pretreated with autophagy inhibitors CQ (10 μM) for 1 h, then treated with quercetin (25 μM for MV4-11, 50 μM for HL-60) for 24 h, and cell death was determined by PI staining and flow cytometry. The data are presented as the mean ± SD; *n* = 3; ***p < 0.001 versus the quercetin group.

It has been reported that quercetin could induce autophagy in various cancer cells, but the autophagy induction by quercetin may provide pro-survival or pro-death mechanism, which may depend on the contexts of cell lines (Ashrafizadeh et al., 2019; Ji et al., 2019). In order to elucidate the possible effect of autophagy in anti-cancer effect of quercetin in MV4-11 cells, we conducted the following experiments. First, we checked the changes of the microtubule-associated protein 1 light chain 3 (LC3) conversion and p62 protein level by western blot. P62 (also known as SQSTM1) is a primary hallmark of autophagy, which interacts with the autophagic effector protein LC3 and subsequently is degraded through an autophagosome-lysosome pathway (Pankiv et al., 2007). As shown in Figure 6A, quercetin treatment resulted in decrease of p62 level, which could be blocked by the presence of chloroquine (CQ, a lysosome inhibitor), accompanied by the downregulation of mTORC1 signaling proteins. As mTORC1 signaling plays an important negative regulatory role in both autophagic induction and lysosome function (Settembre et al., 2012; Zhou et al., 2013), we further detected whether quercetin could affect lysosome function. As shown in Figure 6B, LysoTracker staining was increased by quercetin in MV4-11 and HL-60 cells, indicating enhanced acidification and lysosomal activation. Our data suggested that suppression of mTORC1 activity by quercetin promotes autophagic induction and lysosome activation. To further determine whether there is any relationship between quercetin-induced apoptosis and autophagy in AML cells, apoptosis was detected after exposure to quercetin with or without either CQ (an autophagic inhibitor). As shown in Figure 6C, combined treatment with CQ caused more cell death than quercetin alone treatment in both MV4-11 and HL-60 cells. These results collectively suggested that quercetin-mediated autophagy induction confers protection from quercetin-induced apoptosis.

## Discussion

**FIGURE 7:**
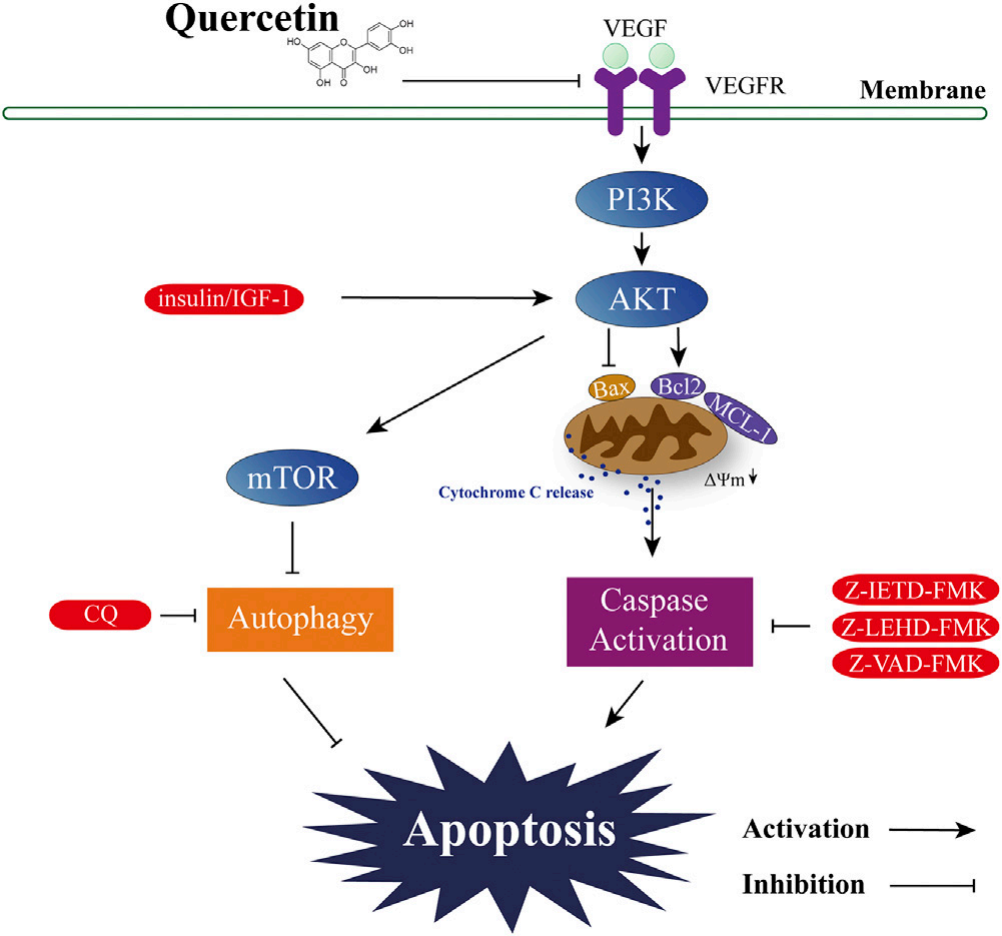
Proposed signal transduction pathways by which quercetin induces apoptosis in AML cells.

Although the anti-cancer property of quercetin has been studied in multiple types of solid tumor, its effect on AML is less studied, and the underlying mechanisms still largely unknown. In this study we provided compelling evidence showing that downregulation of VEGF-Akt signaling is the key mechanism for quercetin-induced apoptotic cell death; furthermore, we identified the involvement of Bcl-2 proteins and mitochondrial membrane potential acting as downstream of VEGF-Akt signaling. Therefore, results from this study revealed a novel mode of action for the cytotoxic activity of quercetin: downregulation of VEGF-Akt signaling leads to caspase-dependent apoptotic cell death, accompanied by altered expressions of Bcl-2 family proteins and depolarization of mitochondrial membranes, which facilitates the release of cytochrome C and thus amplifies the caspase cascade (Figure 7).

We first confirmed the effect of quercetin in apoptotic induction in AML cells, basing on the following observations: 1) the typical apoptotic cell death in AML cell after quercetin treatment, which can be reversed by both pan-caspase inhibitor and specific caspase inhibitors, specific for caspase 8 and caspase 9 (Figures 2B–D); 2) the dose-dependent cleavage of caspase proteins and PARP (Figure 2A), another hallmark for apoptotic cell death. More importantly, quercetin inhibits leukemic cell growth but has little toxicity on normal monocytes (Supplementary Figure S1), which greatly enhance the possible application of quercetin in AML therapy. All these findings are consistent with previous quercetin studies observed in other cancer cell lines (Larocca et al., 1991; Liu et al., 2017). As reported previously, quercetin could act on various aspects of cell proliferation and cell death to exert its antitumor function in hematologic malignancies, such as targeting at MAPK signaling, and cell cycle profile (Maso et al., 2014; Calgarotto et al., 2018). However, the interlinks and upstream regulators of these events are still largely unknown.

The most important finding in our study is the establishment of inhibitory effects on VEGF/Akt signaling by quercetin in AML cells. AML is the most common acute leukemia diagnosed, and the majority of patients with AML die from disease relapse. VEGFR2 signaling regulates mitochondrial biogenesis and metabolism of AML, and provide survival advantage and chemotherapeutic resistance for AML (Nobrega-Pereira et al., 2018). In this study, quercetin was observed to reduce the level of VEGFR2 in MV4-11 cells and inhibit the secretion of VEGF in culture medium, suggesting the blockade of the VEGF signaling by quercetin. According to the key role of VEGF in angiogenesis, this signaling pathway has been implicated as the central mediator of tumor neovascularization, and the interaction between VEGF and VEGFR2 has been demonstrated to be correlated to migration and differentiation of various tumor cells (Serrano et al., 2007; Jeanne and Jayesh, 2012). Expression level of VEGFR2 has been associated with decreased remission rates and reduced survival in various cancers (Weis and Cheresh, 2011). Previous studies suggested that anti-VEGF antibody has been used in combination with conventional chemotherapy in the treatment of refractory and relapsed AML (Madlambayan et al., 2010). Consistently, when sorafenib (a well-known VEGFR inhibitor) was applied on MV4-11 cells, we also observed significantly cell death and decreased cell viability (Supplementary Figure S2), indicating the feasible application of quercetin as therapeutic approach for AML treatment by targeting VEGF/VEGFR2.

Activated VEGFR2 has been reported to mediate the phosphorylation of many proteins, such as Akt and MAPK (Gong et al., 2019). Quercetin had been demonstrated to induce apoptosis via ROS-mediated MAPK activation (Lee et al., 2015). However, we did not observe any ROS change after quercetin treatment in MV4-11 and HL-60 cells (data not shown), but did detect the dose-dependent decrease of both ERK and JNK phosphorylation (Supplementary Figure S3) which is controversial to previous reports (Lee et al., 2017). This discrepancy on ERK activation may be explained by the quercetin-mediated VEGFR2 inhibition in MV4-11 cells. Unfortunately, we failed to provide further experimental evidence to confirm the involvement of MAPK in quercetin-induced cell death in two AML cell lines. It will be interesting to do further investigation on the MAPK proteins in our future study.

On the other hand, another VEGFR2 downstream factor, Akt is critical for delivering the effect of VEGFR2 inhibition to mitochondrial apoptosis after quercetin treatment, based on the following observations: 1) proteomic data showed that the expression levels of Akt and its downstream proteins were downregulated (Table 1); 2) quercetin is able to decrease the level of anti-apoptotic Bcl-2 proteins (Bcl-2 and Mcl-1), following the reduction of Akt phosphorylation in MV4-11 cells; 3) Consistently, insulin, IGF-1 and VEGF can partially block the reduction of Akt phosphorylation, and rescue the quercetin-induced cell death. It has been well established that Bcl-2 family members are downstream factors of Akt signaling, and they are also critical mediators relaying the death signal from the initiator caspase 8 to caspase 9 and eventually the effector caspase 3 and apoptotic cell death (Green and Llambi, 2015). Our findings revealed that the regulatory effect on Bcl-2 anti-apoptotic proteins and mitochondrial apoptosis by quercetin is due to the downregulation of VEGFR2/Akt signaling. It will be important to confirm this finding with *in vivo* model in our future work.

Taking together, our findings from this study provide convincing evidence that the suppression on VEGF/VEGFR2 by quercetin relays the cell death signaling from Akt/Bcl-2 signaling to mitochondria, and eventually leading to caspase-dependent cell death. Understanding the molecular mechanisms underlying quercetin-induced apoptosis in AML cells will provide new insights for further investigation and development of quercetin into a potential anticancer agent.

## Data Availability

The original contributions presented in the study are included in the article/Supplementary Material, further inquiries can be directed to the corresponding authors.
