# Corrigendum: Quercetin Induces Apoptosis Via Downregulation of VEGF/Akt Signaling Pathway in Acute Myeloid Leukemia Cells

**DOI:** 10.3389/fphar.2020.640750

**Published:** 2021-02-08

**Authors:** Huan Shi, Xin-Yu LI, Yao Chen, Xing Zhang, Yong Wu, Zi-Xuan Wang, Pan-Hong Chen, Hui-Qi Dai, Ji Feng, Sayantan Chatterjee, Zhong-Jie Li, Xiao-Wei Huang, Hong-Qiao Wei, Jigang Wang, Guo-Dong Lu, Jing Zhou

**Affiliations:** ^1^ Department of Physiology, School of Preclinical Medicine, Guangxi Medical University, Nanning, China; ^2^ Department of Physiology, School of Medicine, Hunan University of Medicine, Huaihua, China; ^3^ Artemisinin Research Center and the Institute of Chinese Materia Medica, China Academy of Chinese Medical Sciences, Beijing, China; ^4^ Department of Toxicology, School of Public Health, Guangxi Medical University, Nanning, China; ^5^ Key Laboratory of High-incidence-Tumor Prevention and Treatment (Guangxi Medical University), Ministry of Education of China, Nanning, China; ^6^ Cancer Science Institute of Singapore, National University of Singapore, Singapore, Singapore

**Keywords:** quercetin, vascular endothelial growth factor/PI3K/Akt, mitochondria, apoptosis, acute myeloid leukemia

In the published article, the author list was missing the signs and an indication. The correct authors list is “Huan Shi^1,2,†^, Xin-Yu Li^1,†^, Yao Chen^1,†^, Xing Zhang^3,†^, Yong Wu^4^, Zi-Xuan Wang^4^, Pan-Hong Chen^1^, Hui-Qi Dai^4^, Ji Feng^4^, Sayantan Chatterjee^1^, Zhong-Jie Li^1^, Xiao-Wei Huang^4^, Hong-Qiao Wei^1^, Jigang Wang^3^, Guo-Dong Lu^4,5,6*^ and Jing Zhou^1*^” and it should also include one statement “^†^These authors contributed equally to this work” as an indication for “†” in the author list in the published article.

The authors apologize for this error and state that this does not change the scientific conclusions of the article in any way. The original article has been updated.

