# Supplementary material for: Quercetin Induces Apoptosis via Downregulation of Vascular Endothelial Growth Factor/Akt Signaling Pathway in Acute Myeloid Leukemia Cells
Source: Front Pharmacol. 2020 Dec 10;11:534171. doi: 10.3389/fphar.2020.534171 (PMC7758733; doi:10.3389/fphar.2020.534171)
Supplement: Supplementary file 1 [file datasheet1.pdf]

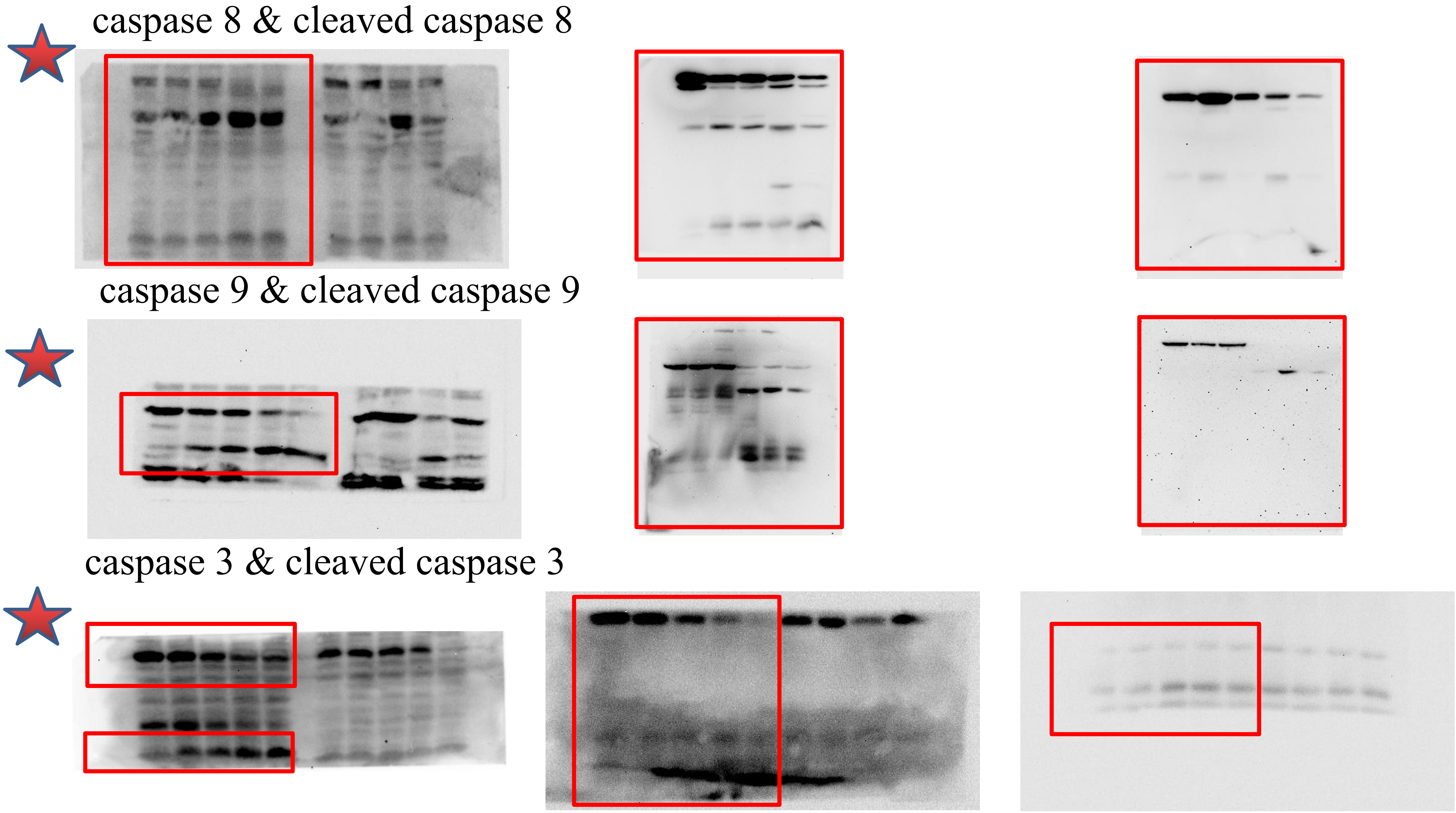
caspase 8 & cleaved caspase 8
caspase 9 & cleaved caspase 9
caspase 3 & cleaved caspase 3

Figure 2A

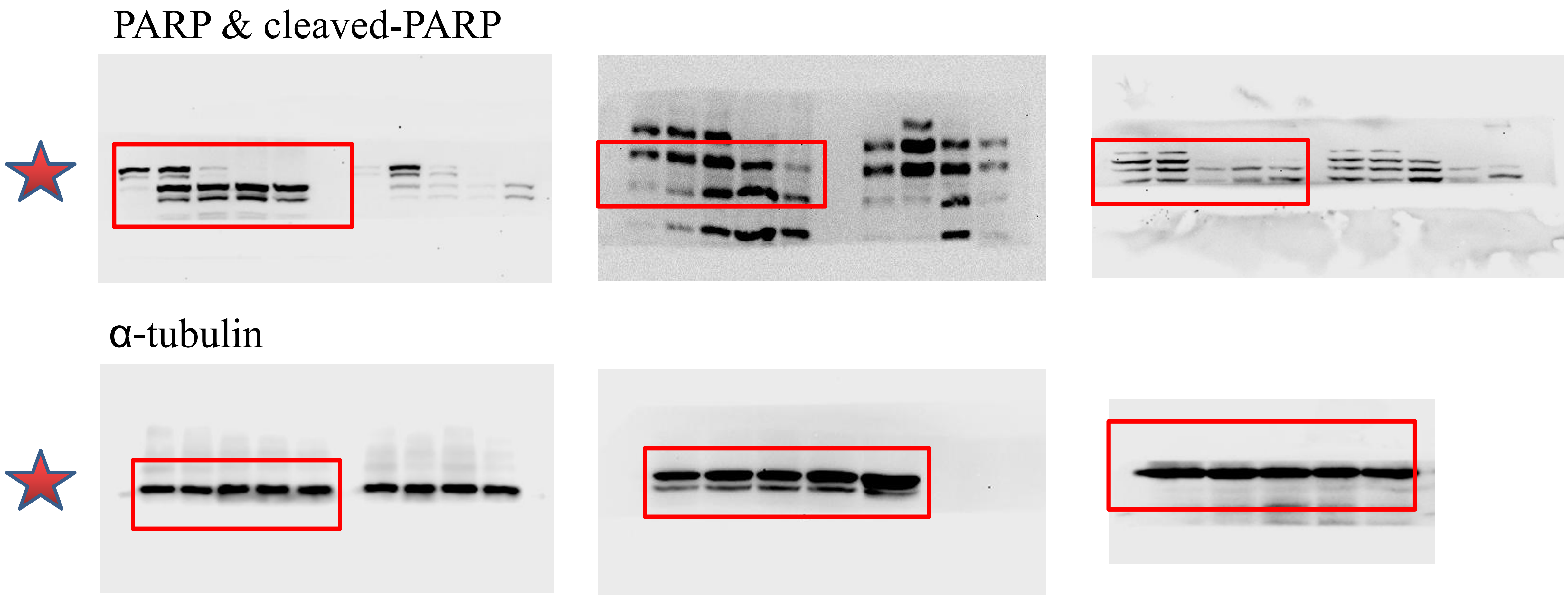

Figure 2A

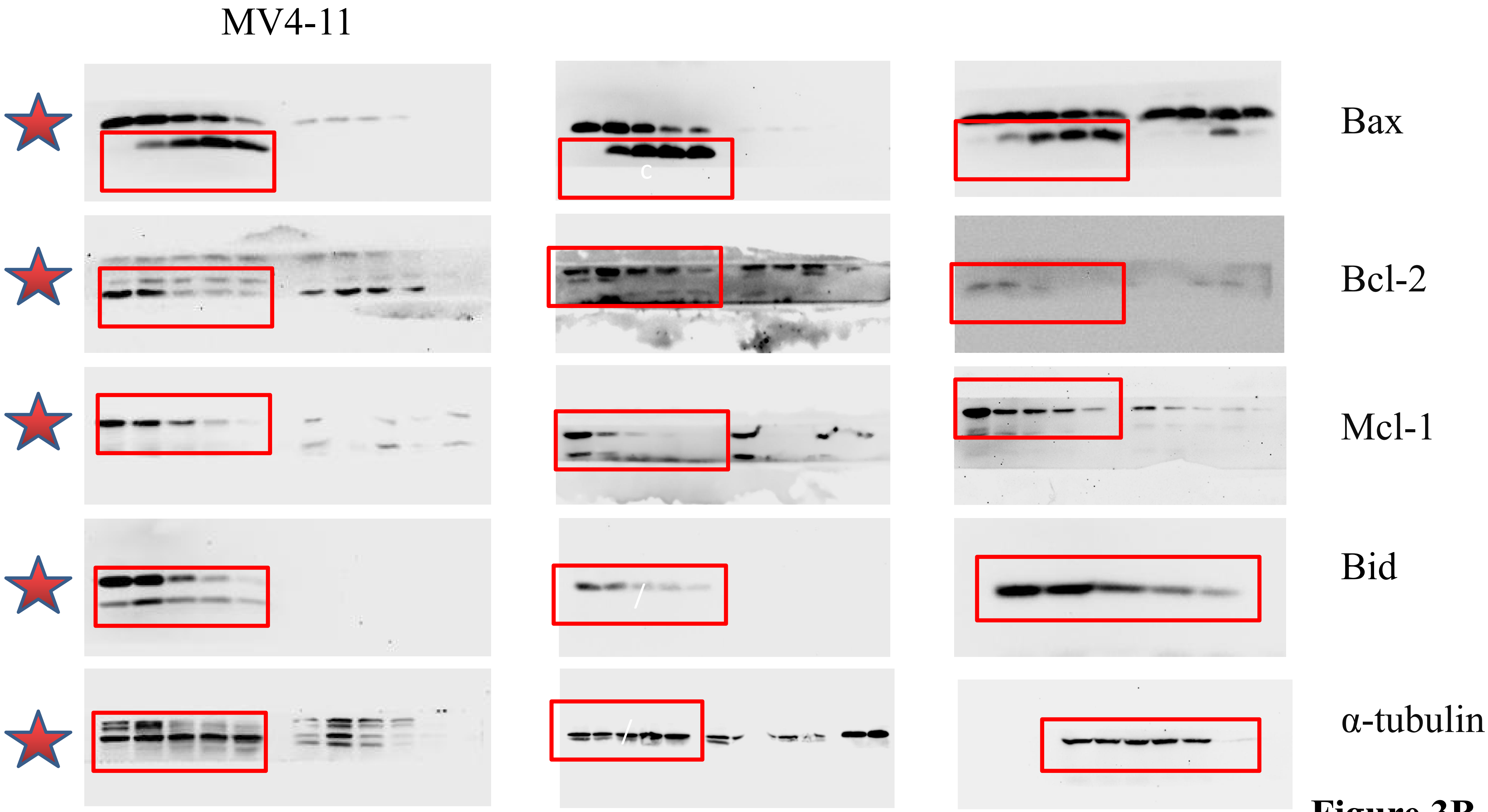

Figure 3B（1）

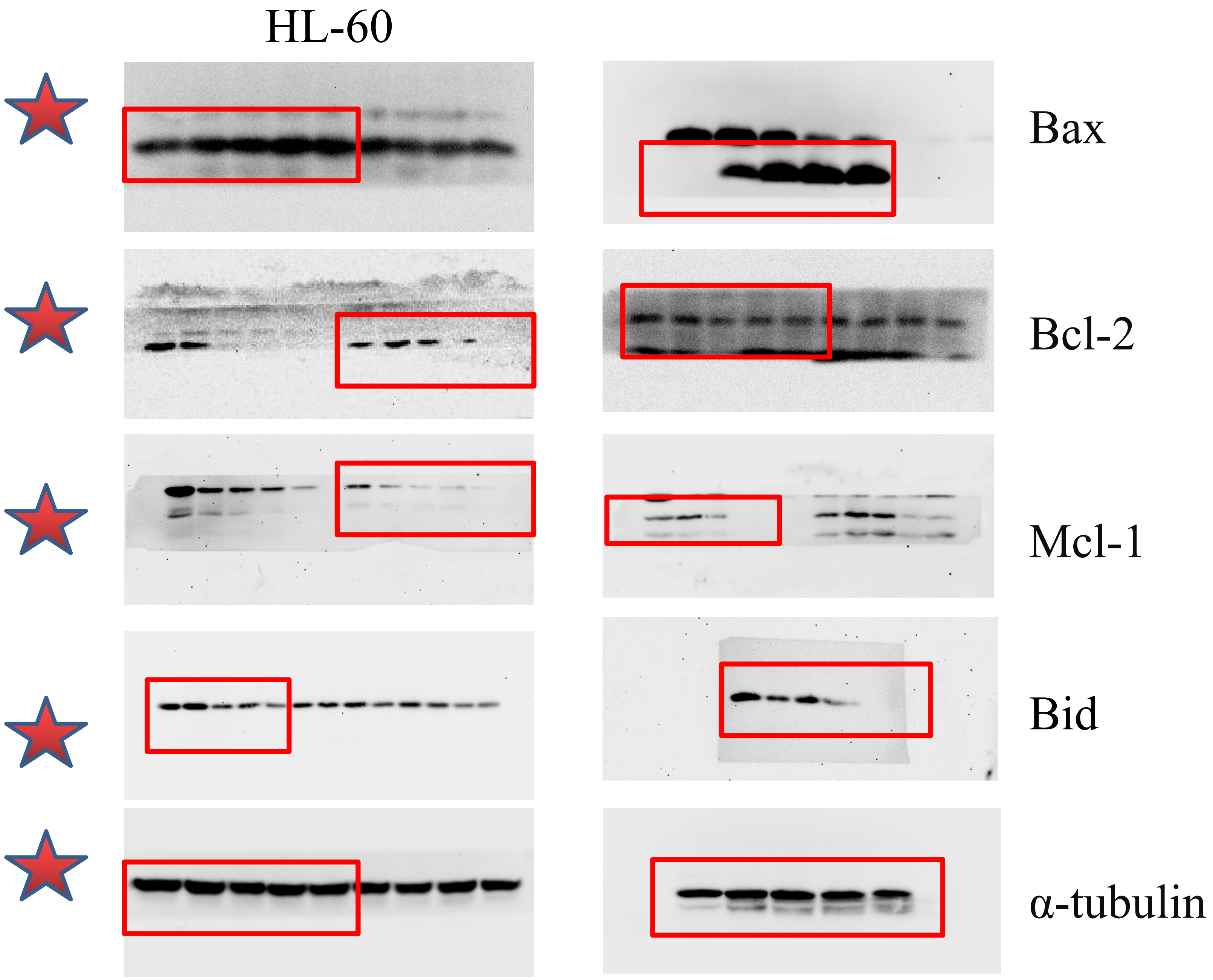

Figure 3B（2）

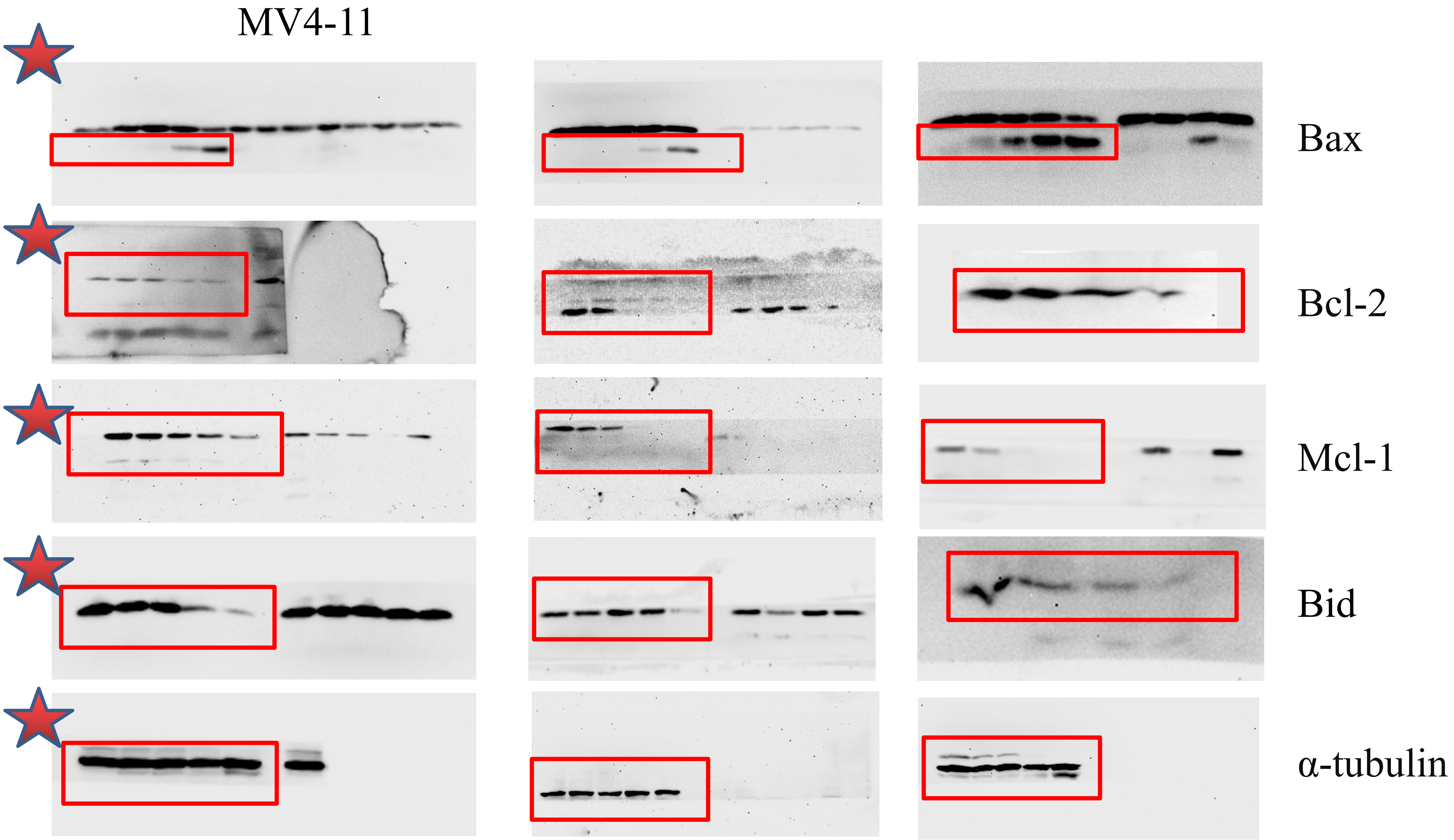

Figure 3B（3）

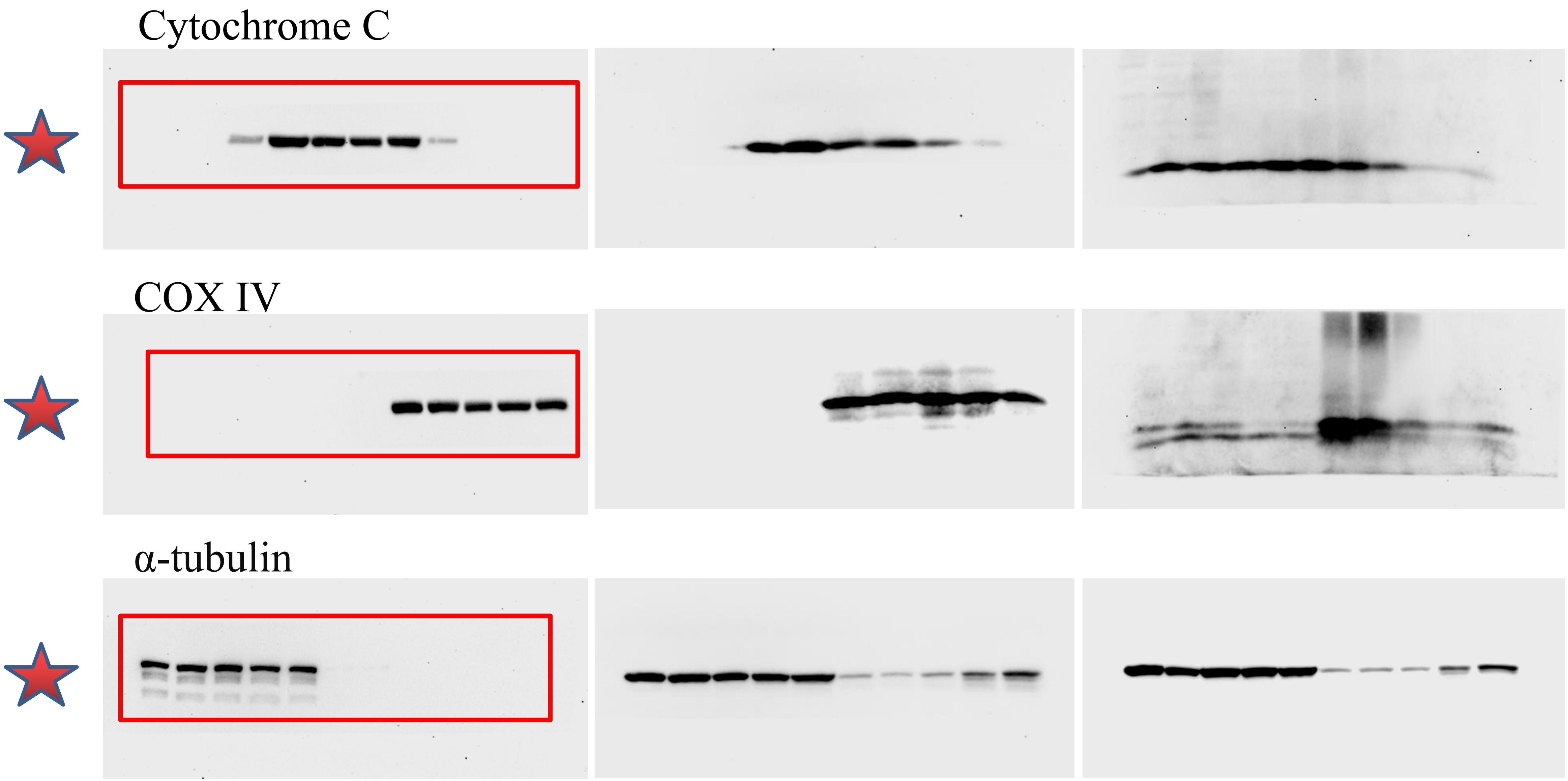

Figure 3C

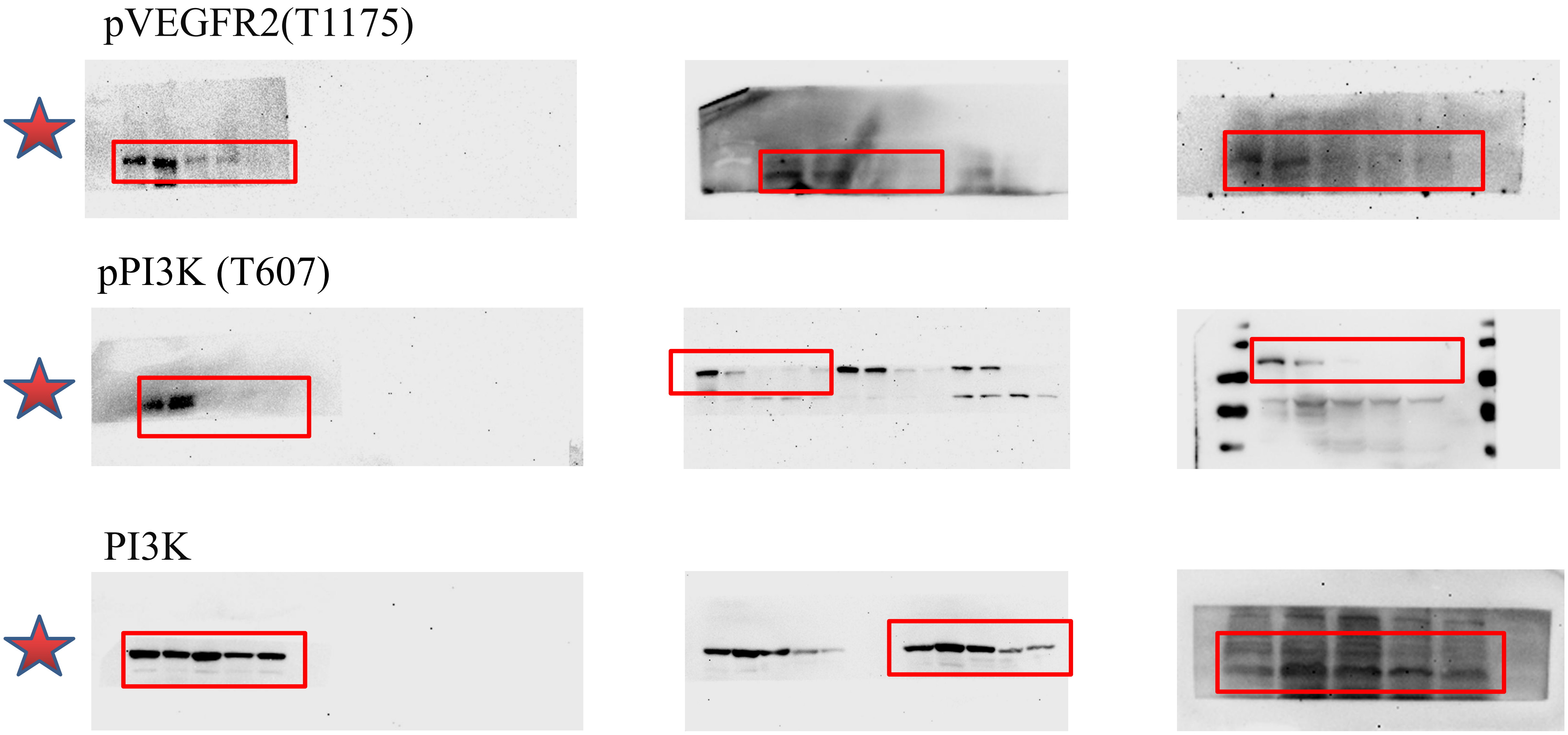

Figure 4B

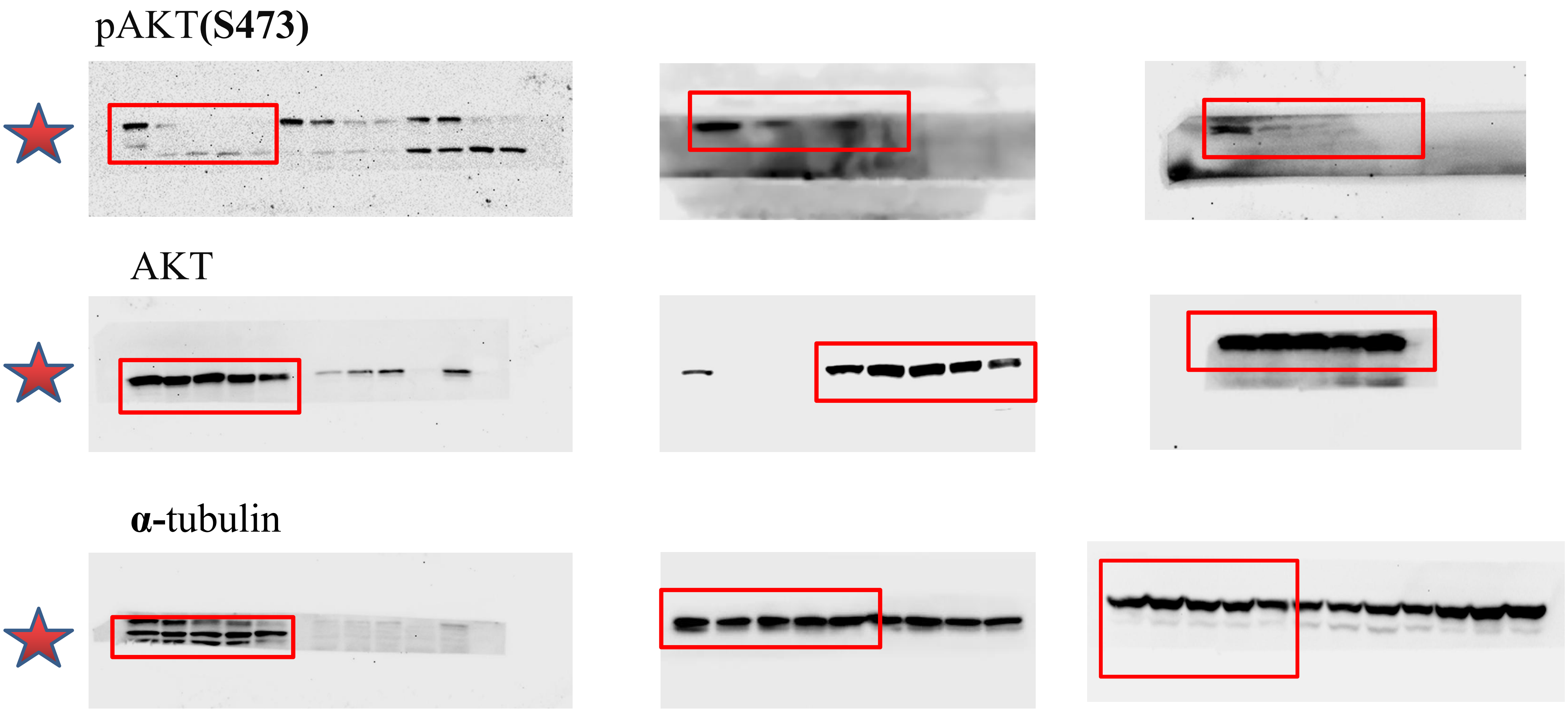

Figure 4B

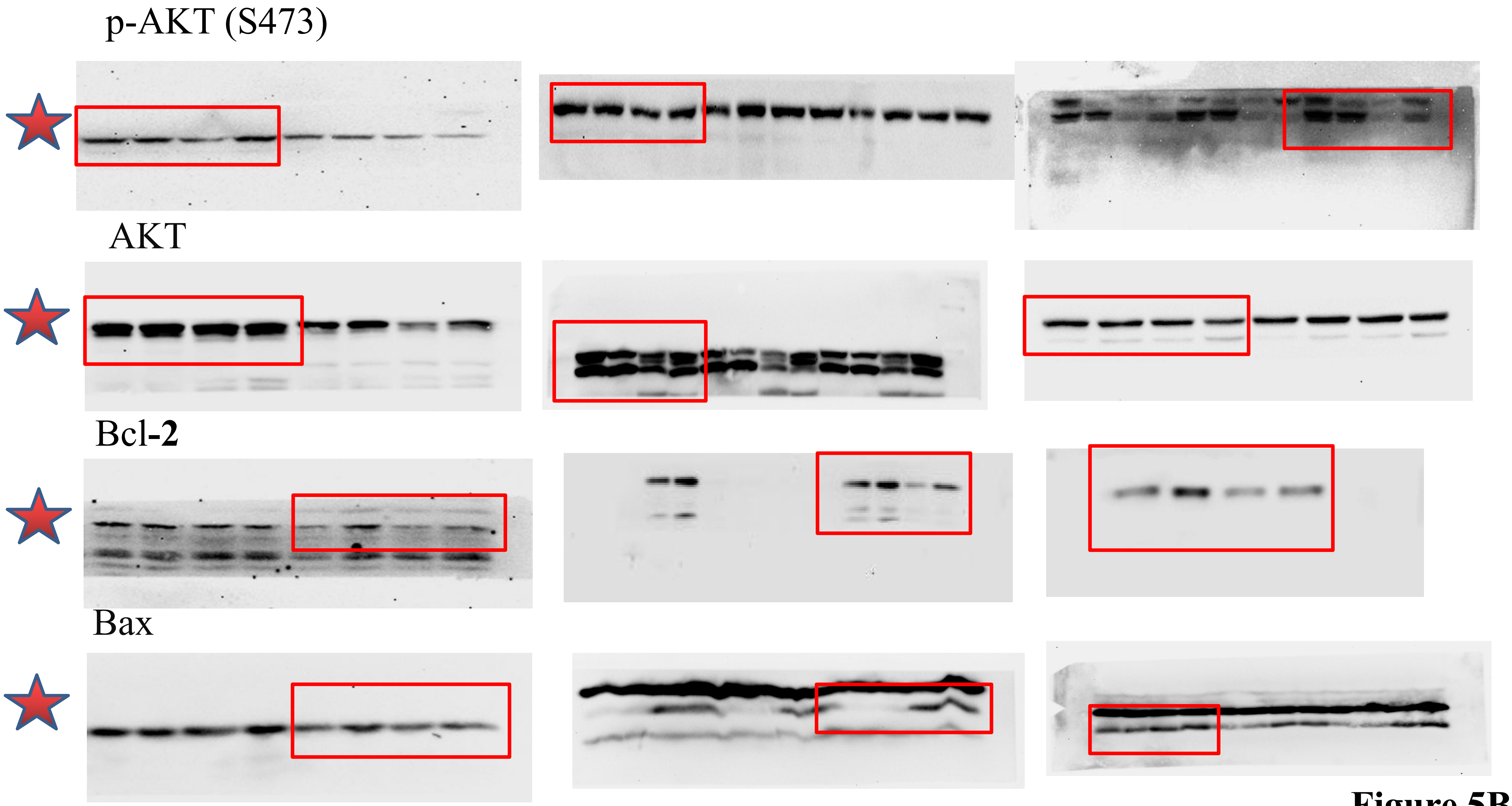

Figure 5B

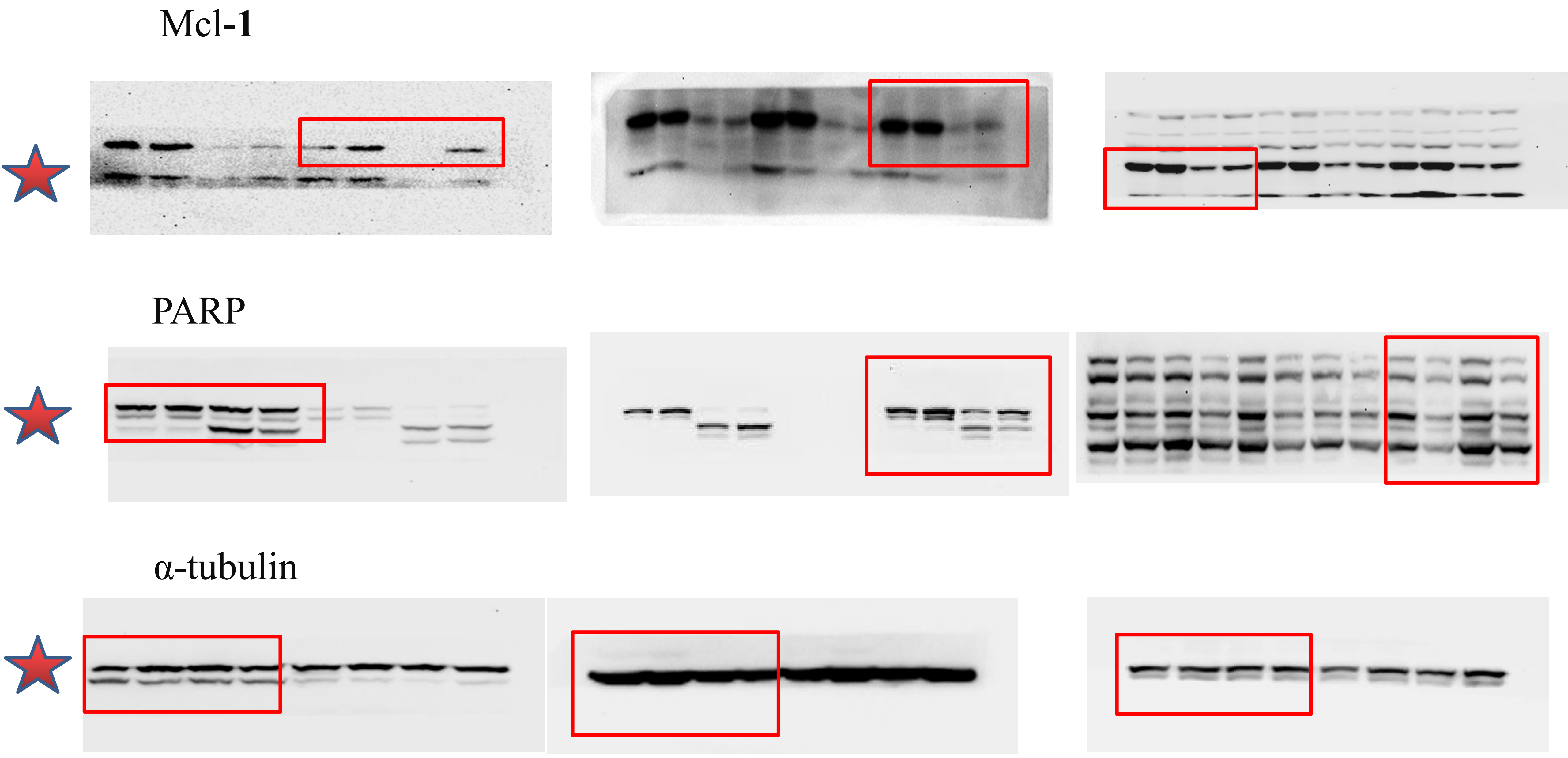

Figure 5B

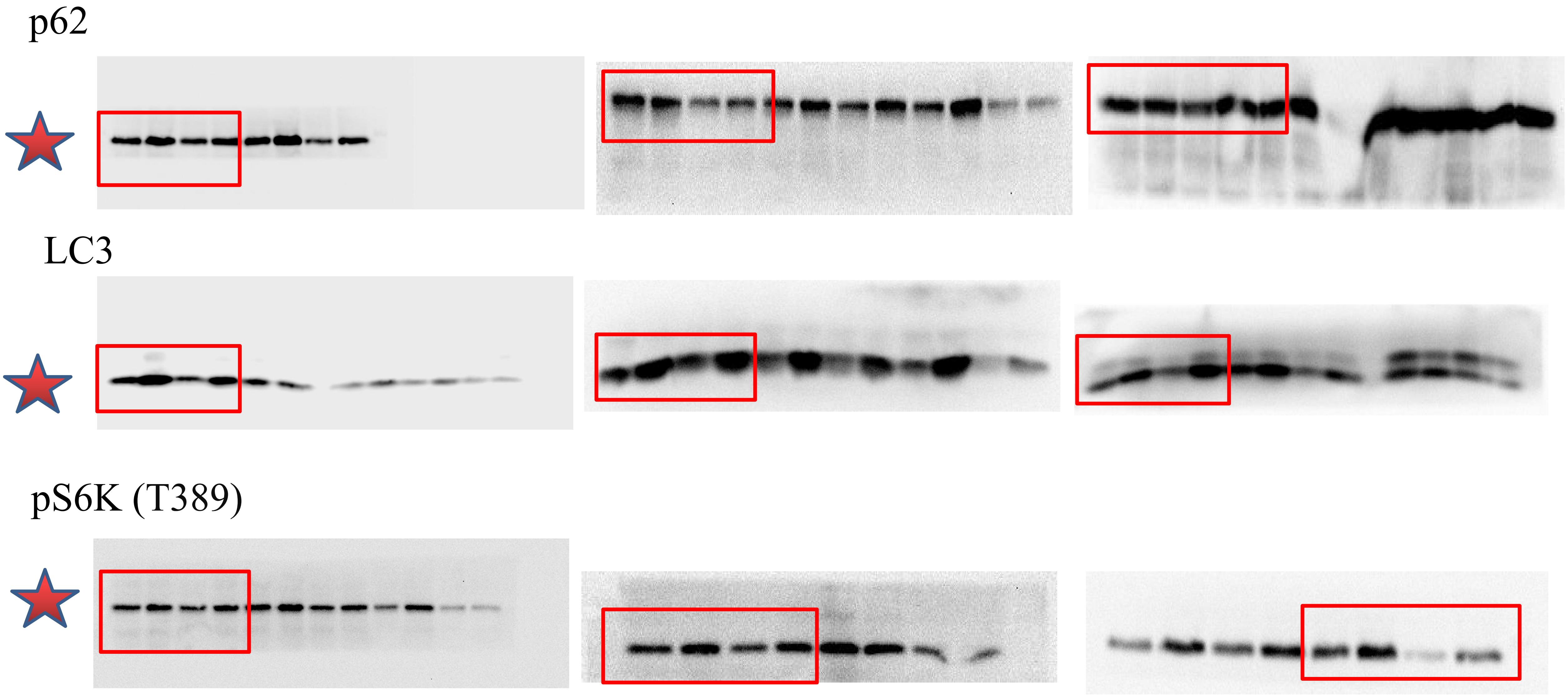

Figure 6A

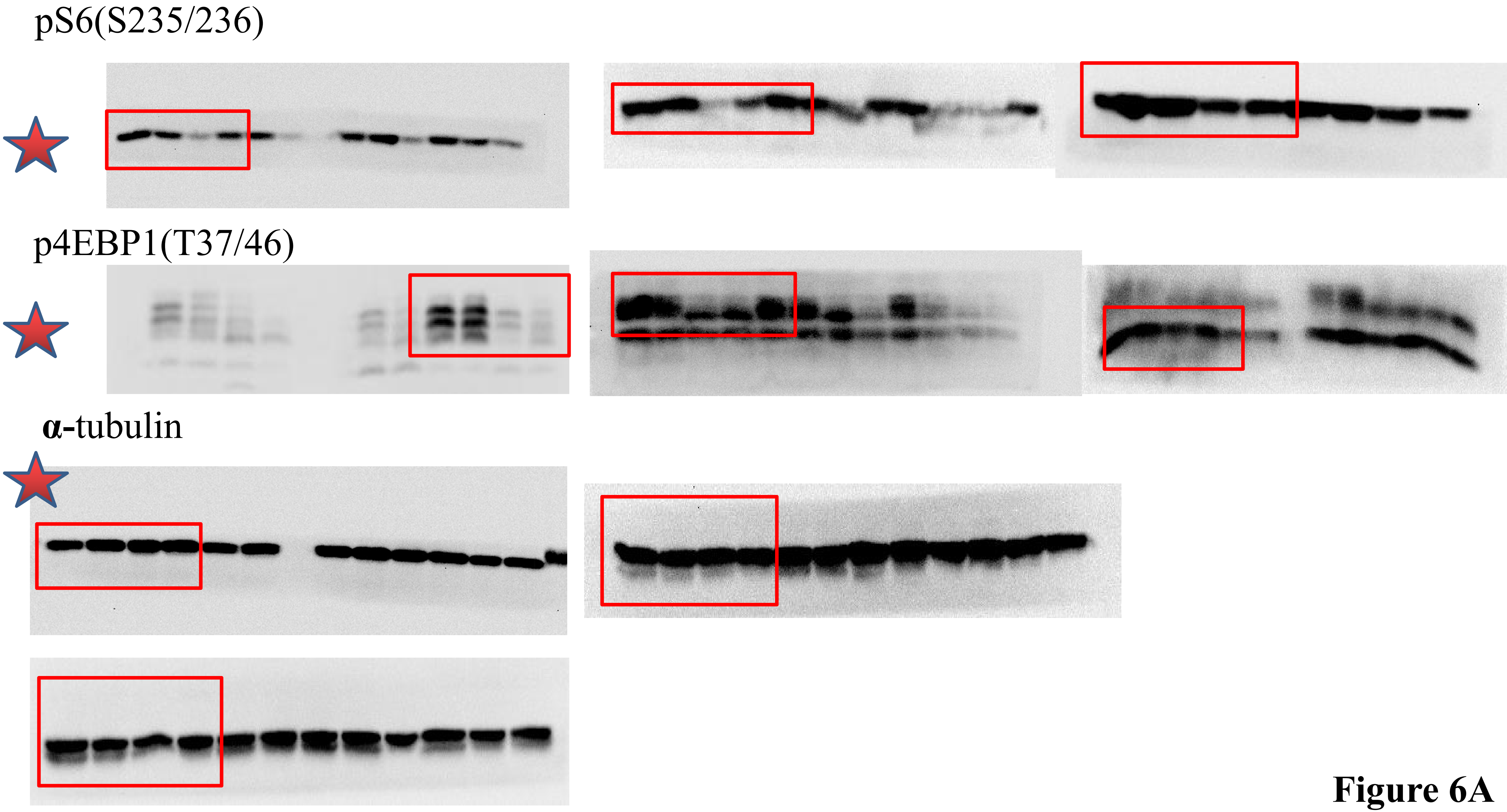

Figure 6A

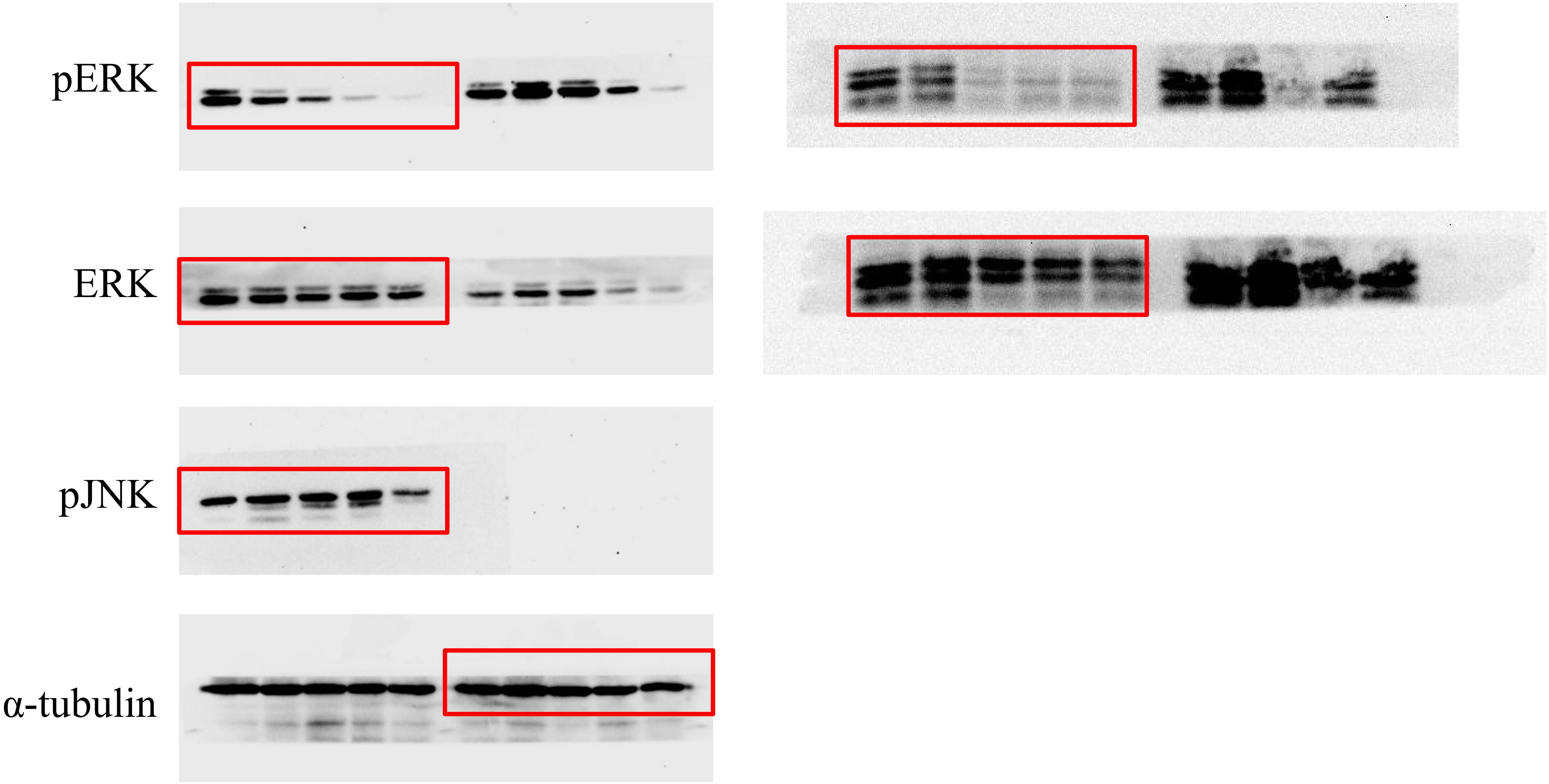

Supplementary figure 3
