# Supplementary material for: Quercetin Induces Apoptosis via Downregulation of Vascular Endothelial Growth Factor/Akt Signaling Pathway in Acute Myeloid Leukemia Cells
Source: Front Pharmacol. 2020 Dec 10;11:534171. doi: 10.3389/fphar.2020.534171 (PMC7758733; doi:10.3389/fphar.2020.534171)
Supplement: Supplementary file 2 [file datasheet2.pdf]

Supplementary Material

1.1 Supplementary Figures Legends

Supplementary Figure 1. Quercetin has little toxicity on normal monocytes.

(A) Human peripheral blood mononuclear cells (PBMC) were collected and treated with quercetin at different concentrations for 24 h. After staining with Propidium Iodide (PI), PI-positive cells were counted by flow cytometer. **(B)** Cells were treated as indicated in panel (A), then cell death was assessed by LDH release assay. Ratios of LDH activity in individual group were normalized by control group. The data are presented as the mean ± SD, n=3, ns: not significant; $p>0.05$ versus the control group.

Supplementary Figure 2. Sorafenib significantly induces cell death and decreases MV4-11 cells viability.

(A) Effects of various doses of sorafenib on cell viability of MV4-11. Cells were seeded in 96 well plates, then treated with indicated concentrations of sorafenib for 24 h, Cell Counting Kit-8 (CCK8) was added 3 h before termination of the experiments and the absorbance was measured at 450 nm. **(B)** MV4-11 cells were treated with quercetin at different concentrations for 24 h. After staining with PI, and PI-positive cells were counted by flow cytometer for the indicated concentrations. The data are presented as the mean ± SD; n=3, ** $p<0.01$, *** $p<0.001$ versus the control (0 μM) group.

Supplementary Figure 3. Quercetin reduces the phosphorylation level of ERK and JNK.

MV4-11 cells were treated with quercetin for indicated concentrations, then cell lysates were collected and subjected to western blotting with the indicated antibodies.

Supplementary Figure 4. Quercetin induces LDH release in MV4-11 and HL-60 cells.

MV4-11 and HL-60 cells were treated with indicated concentrations of quercetin for 24 h, and cell death was assessed by LDH release assay, and ratios of LDH activity of individual group were normalized by control are shown. The data are presented as the mean ± SD, n=3, *** $p<0.001$ versus the control group.
