# Supplementary material for: Quercetin Induces Apoptosis via Downregulation of Vascular Endothelial Growth Factor/Akt Signaling Pathway in Acute Myeloid Leukemia Cells
Source: Front Pharmacol. 2020 Dec 10;11:534171. doi: 10.3389/fphar.2020.534171 (PMC7758733; doi:10.3389/fphar.2020.534171)
Supplement: Supplementary file 4 [file datasheet4.docx]

**Isobaric Tag for Relative and Absolute Quantification (iTRAQ) Labeling and LCMS/MS Analyses**

The synthesis of quercetin-based analogue and probe was conducted and validated as previously (Wang et al., 2016). Then, a clickable activity-based protein profiling (ICABPP) method was used as well to pull down quercetin probe treated samples. Briefly, MV4-11 cells were treated with quercetin probe or DMSO (as vector control) for 12 or 24hr. Equal amounts of cell lysates (two biological replicates for quercetin probe and two for DMSO) were used for click chemistry to form protein conjugate with biotin tags respectively. The precipitated protein pellets were digested with trypsin (Promega) and the resultant peptides were separated using a filter-spin column (GE Healthcare).

The pull-down samples of quercetin probe were labelled with iTRAQ reagents 116 and 117, while those of DMSO with iTRAQ reagents 113 and 114 (AB SCIEX, Foster City, CA). After all samples were pooled together, the iTRAQ-labelled peptide samples were purified by an iTRAQ Method Development Kit (SCIEX, 4352160) using the strong cation exchange chromatography technique. Then the pooled peptide mixtures were separated using an Eksigent NanoLCUltra system coupled to the ChiPLC-Nanoflex system (Eksigent, USA), before detection by MS/MS with a TripleTOF 5600 system (SCIEX).

Lastly, the ProteinPilot^TM^ Software 4.5 (SCIEX) coupled with ParagonTM algorithm were selected to conduct database searches, in order to identify and quantify the relevant proteins. The database includes a total of 91,468 human protein sequence entries according to the International Protein Index v3.87. The function of Progroup algorithm was used to eliminate redundancy, while a decoy database search to determine the false discovery rate. The data were finally exported to an Excel file for manual data analysis. The cutoff thresholds were set at >1.3 or <0.77.
