# Supplementary figures and images for: Quercetin Induces Apoptosis via Downregulation of Vascular Endothelial Growth Factor/Akt Signaling Pathway in Acute Myeloid Leukemia Cells
Source: Front Pharmacol. 2020 Dec 10;11:534171. doi: 10.3389/fphar.2020.534171 (PMC7758733; doi:10.3389/fphar.2020.534171)

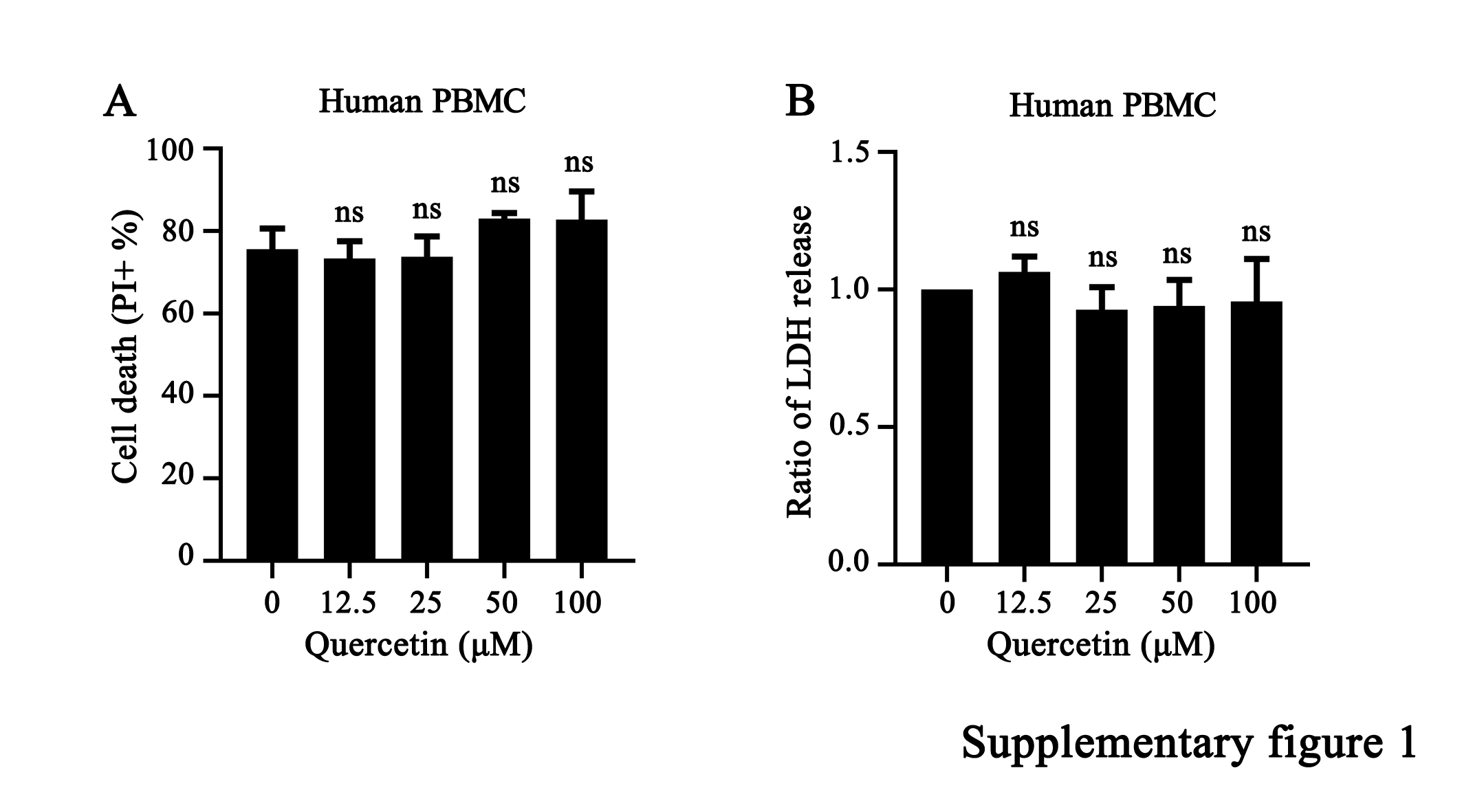

Supplement: Supplementary file 5 [file image1.tif]

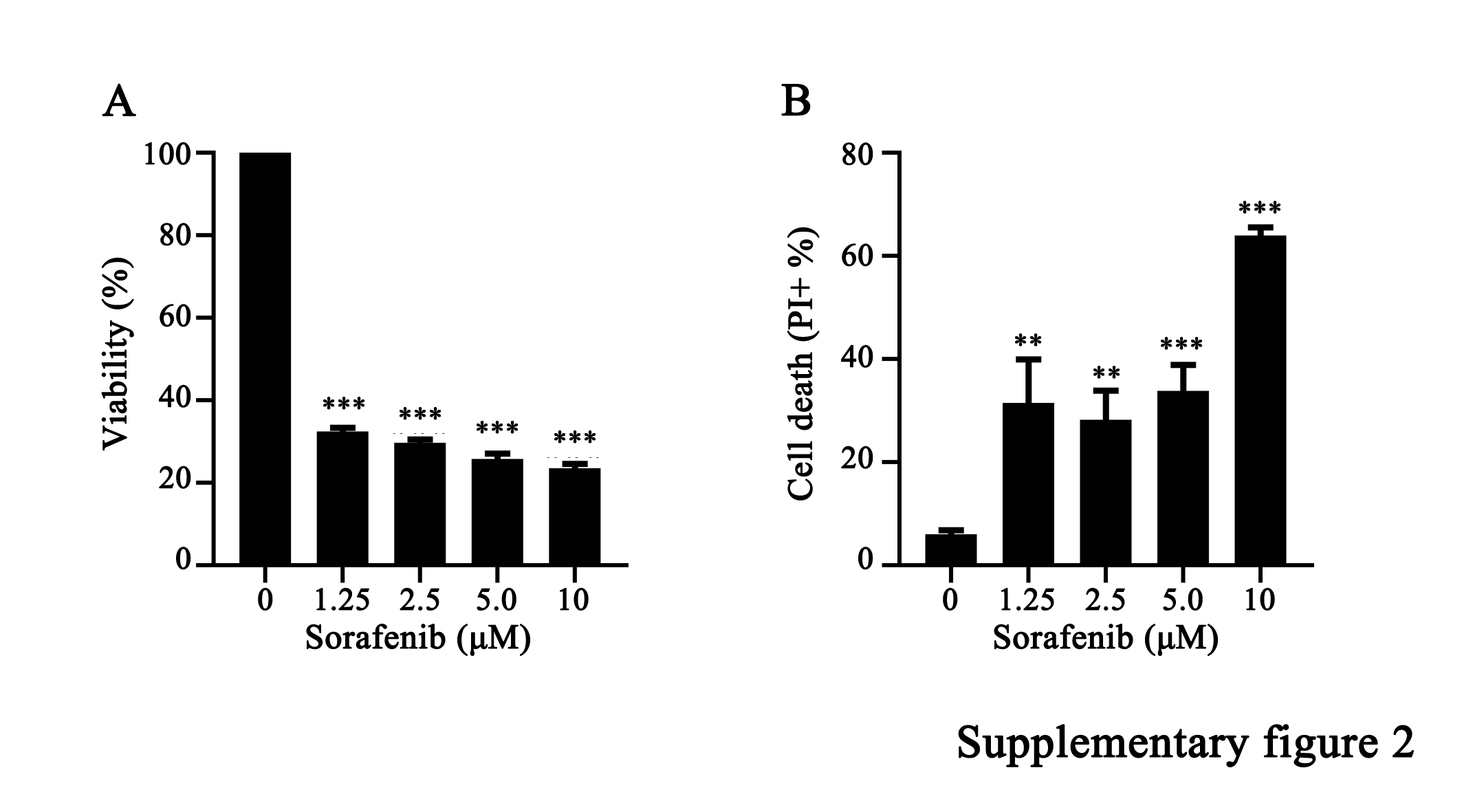

Supplement: Supplementary file 6 [file image2.tif]

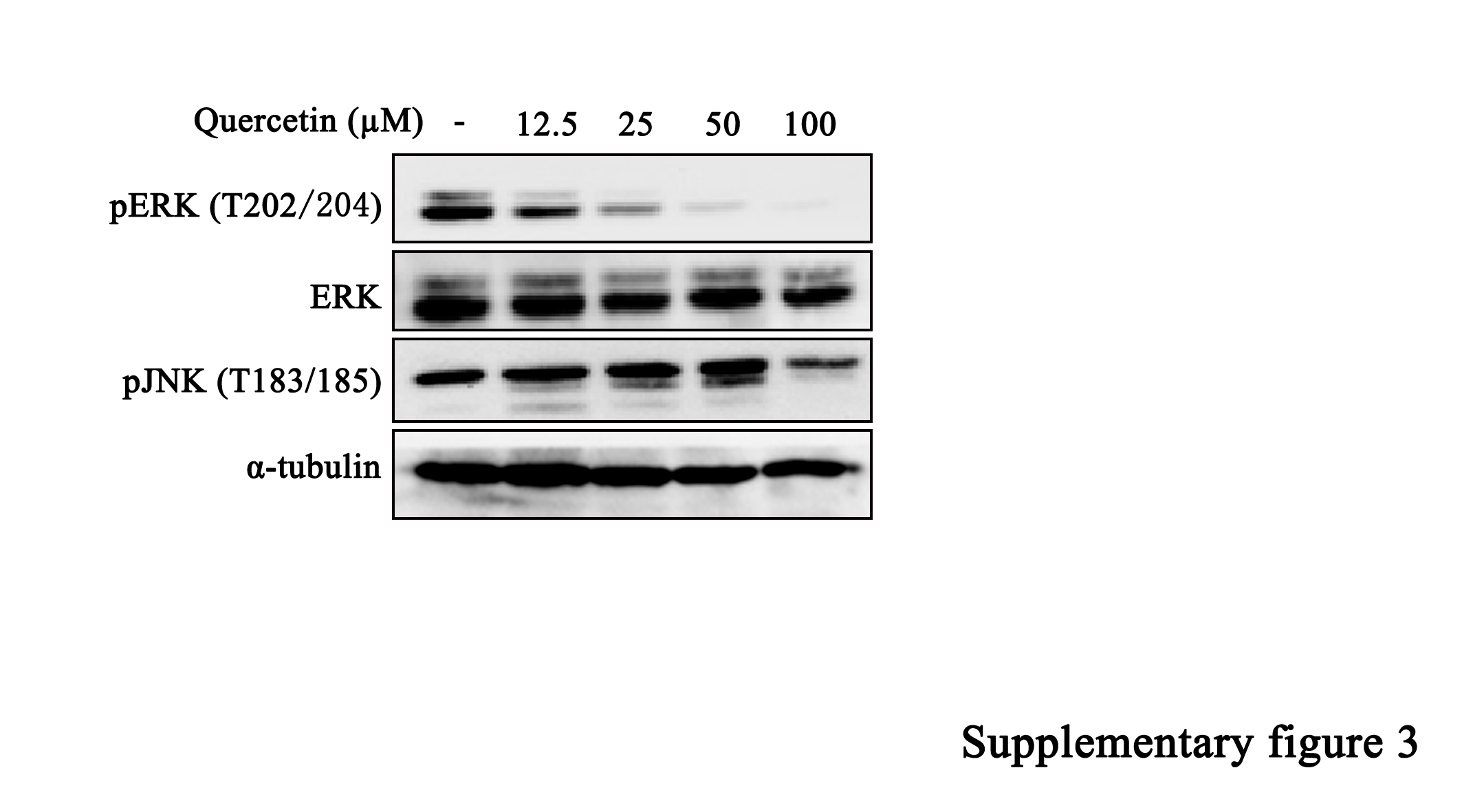

Supplement: Supplementary file 7 [file image3.tif]

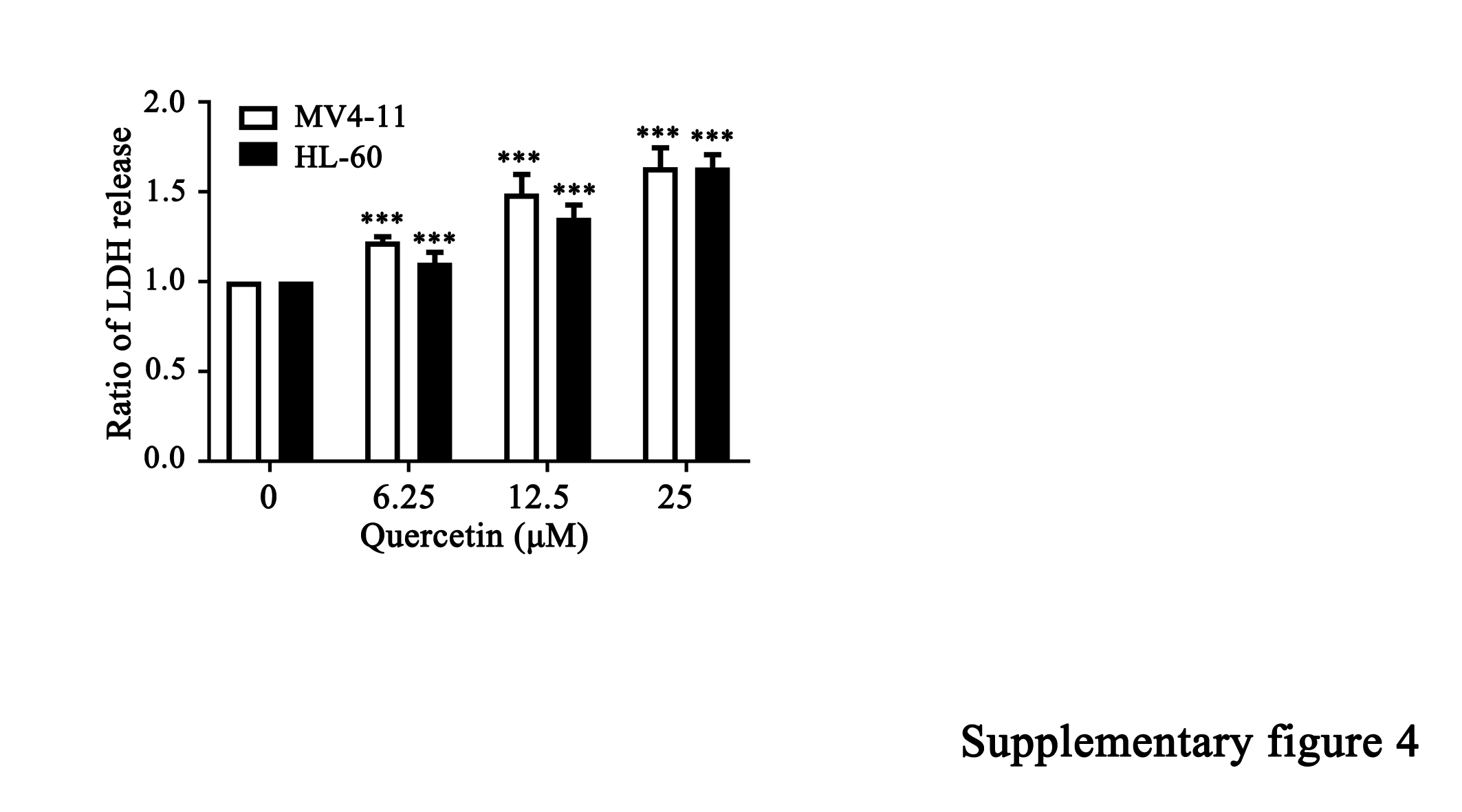

Supplement: Supplementary file 8 [file image4.tif]
